# Highly Efficient Photosensitizers with Molecular Vibrational Torsion for Cancer Photodynamic Therapy

**DOI:** 10.1021/acscentsci.3c00611

**Published:** 2023-07-17

**Authors:** Xiao Zhou, Chao Shi, Saran Long, Qichao Yao, He Ma, Kele Chen, Jianjun Du, Wen Sun, Jiangli Fan, Bin Liu, Lei Wang, Xiaoqiang Chen, Laizhi Sui, Kaijun Yuan, Xiaojun Peng

**Affiliations:** † State Key Laboratory of Fine Chemicals, Dalian University of Technology, 2 Linggong Road, Dalian 116024, P. R. China; ‡ State Key Laboratory of Fine Chemicals, College of Materials Science and Engineering, Shenzhen University, Shenzhen 518060, P. R. China; § College of Chemistry and Chemical Engineering, Yantai University, Yantai 264005, P. R. China; ∥ State Key Laboratory of Molecular Reaction Dynamics, Dalian Institute of Chemical Physics, Chinese Academy of Sciences, Dalian 116023, P. R. China

## Abstract

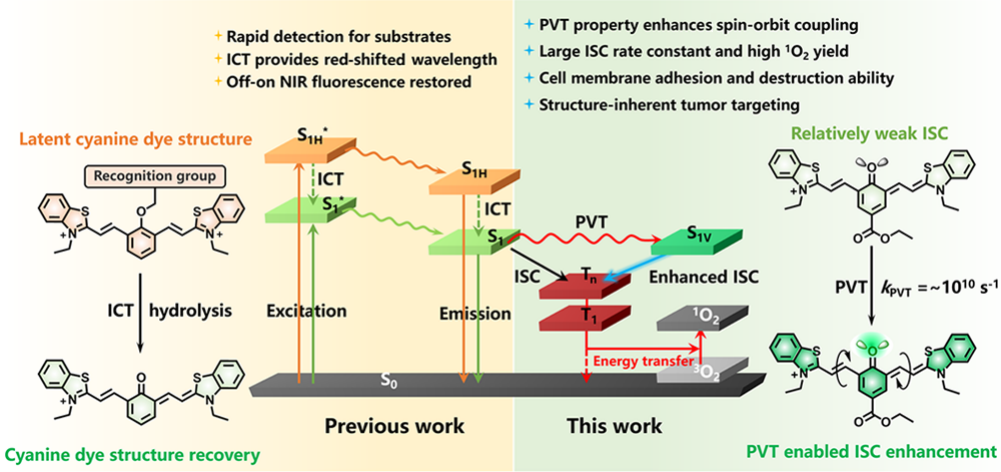

The development of highly effective photosensitizers (PSs) for photodynamic therapy remains a great challenge at present. Most PSs rely on the heavy-atom effect or the spin–orbit charge-transfer intersystem crossing (SOCT-ISC) effect to promote ISC, which brings about additional cytotoxicity, and the latter is susceptible to the interference of solvent environment. Herein, an immanent universal property named photoinduced molecular vibrational torsion (PVT)-enhanced spin–orbit coupling (PVT-SOC) in PSs has been first revealed. PVT is verified to be a widespread intrinsic property of quinoid cyanine (QCy) dyes that occurs on an extremely short time scale (10^–10^ s) and can be captured by transient spectra. The PVT property can provide reinforced SOC as the occurrence of ISC predicted by the El Sayed rules (^1^ππ*–^3^nπ*), which ensures efficient photosensitization ability for QCy dyes. Hence, QTCy7-Ac exhibited the highest singlet oxygen yield (13-fold higher than that of TCy7) and lossless fluorescence quantum yield (Φ_F_) under near-infrared (NIR) irradiation. The preeminent photochemical properties accompanied by high biosecurity enable it to effectively perform photoablation in solid tumors. The revelation of this property supplies a new route for constructing high-performance PSs for achieving enhanced cancer phototherapy.

## Abstract 2

Photoinduced molecular vibrational torsion-enhanced spin−orbit coupling (PVT-SOC) in quinoid cyanine dyes has been revealed. This strategy enables cyanine photosensitizers to have enhanced intersystem crossing for cancer photodynamic therapy.

## Introduction

Photodynamic therapy (PDT) has been recognized as an effective mild treatment that is minimally invasive and has high spatiotemporal selectivity for cancers.^1−6^ Among several essential factors of photodynamic therapy, the photosensitizer (PS) is the most important component, which is responsible for generating triplet excited states (T_*n*_) through the intersystem crossing (ISC) process under photoexcitation to produce reactive oxygen species (ROS).^7−14^ Therefore, regulating the excited states of photosensitizers and developing new stimulative ISC strategies have been widely concerned.^15−19^

Cyanine dyes are a series of organic functional materials with excellent properties, including convenient synthesis, large molar extinction coefficients, and adjustable absorption and emission wavelengths from the visible to near-infrared (NIR, >650 nm) regions.^20,21^ In addition, the cationic structure of cyanine dyes allows them to be anchored to cell membranes or mitochondrial membranes, thereby enhancing the cellular uptake and further increasing the accumulation in tumor tissues.^22^ For quinoid cyanine (QCy) dyes, a special class of cyanine dyes, the reaction with the substrate could restore the cyanine-like structure of the molecule, which could enhance the intramolecular charge transfer (ICT) effect and recover the NIR fluorescence signal.^23^ Such a structure has only been used in the field of cancer diagnosis or fluorescence imaging so far, which has limited the further application of these intelligent molecules. Therefore, it is valuable to perform research to develop highly efficient photosensitizers with this cyanine framework.

Currently, the heavy-atom effect is a general approach to enhance ISC efficiency and ^1^O_2_ yield for cyanine photosensitizers by introducing halogen atoms with larger atomic numbers into their skeletons,^24−26^ which works because the spin–orbit coupling (SOC) constant is approximately proportional to *Z*^4^, where *Z* is the atomic number.^16,27^ Nevertheless, the fatal deficiency of this strategy is that it also enhances the ISC of T_1_ → S_0_, resulting in the drastically contracted triplet lifetimes,^28,29^ and besides, the connatural cytotoxicity and poor water solubility caused by heavy atoms also limit their further applications.^30−32^ In order to solve this problem, many strategies have been proposed,^33−35^ among which the spin–orbit charge-transfer ISC (SOCT-ISC) mechanism is a widely used method to enhance the SOC process through charge transfer (CT) and charge recombination (CR).^36^ In this process, the transfer of electrons between the two mutually orthogonal orbitals changes their spin angular momentum, therefore enhancing ISC, which was predicted by the El Sayed rules in the 1960s.^37^ Nonetheless, this mechanism is faced with the challenge of limited efficiency in the environments with different polarity due to the existence of a Marcus inverted region in electron transfer. With the increase of Gibbs energy change (Δ*G*) in the charge transfer process, molecules prefer to proceed via charge recombination to the ground state (CR_S_) rather than to the triplet state (CR_T_) after charge separation (CS), which leads to the decrease of ISC rate.^38^ Therefore, it is urgent to develop novel cyanine photosensitizer strategies to solve these problems.

Hence, according to our research, a photoinduced molecular vibrational torsion (PVT)-enhanced spin–orbit coupling (PVT-SOC)-induced intersystem crossing mechanism in QTCy7 dyes and its contribution to enhance ISC capability have been discovered, which we believe can be a novel approach to construct heavy-atom-free photosensitizers for PDT applications. The PVT-SOC property can provide a reinforced spin–orbit coupling through the formation of the vibrational first singlet excited state (S_1V_) as the occurrence of ISC at a conical intersection point between it and the second triplet excited state (T_2_) with partial ^3^nπ* characteristic predicted by the El Sayed rules (^1^ππ*–^3^nπ*) without the introduction of heavy atoms. The PVT effect can occur rapidly (*k*_PVT_ ∼ 10^10^ s^–1^) in the excited state, and the intersystem crossing rate constant can be easily adjusted via introduction of different electron effect groups. By this means, the singlet oxygen yield (Φ_Δ_) of this series of photosensitizers can be promoted to above 20%. Therein, the introduction of a *para*-ester group extremely heightens this effect, resulting in the highest singlet oxygen yield (Φ_Δ_ = 33.8%). In addition, QTCy7-Ac can quickly destroy the integrity of cell membranes under light irradiation with extremely low cytotoxicity, and the strong tumor targeting ability also improves the photoinhibition efficiency for solid tumors due to the inherent targeting capability of the QTCy7 scaffold. We believe that our discovery not only breaks through the restriction of QCy dyes used only in fluorescence detection but also provides a novel approach for constructing efficient photosensitizers.

## Results and Discussion

### Molecular Design and Synthesis

**Figure 1:**
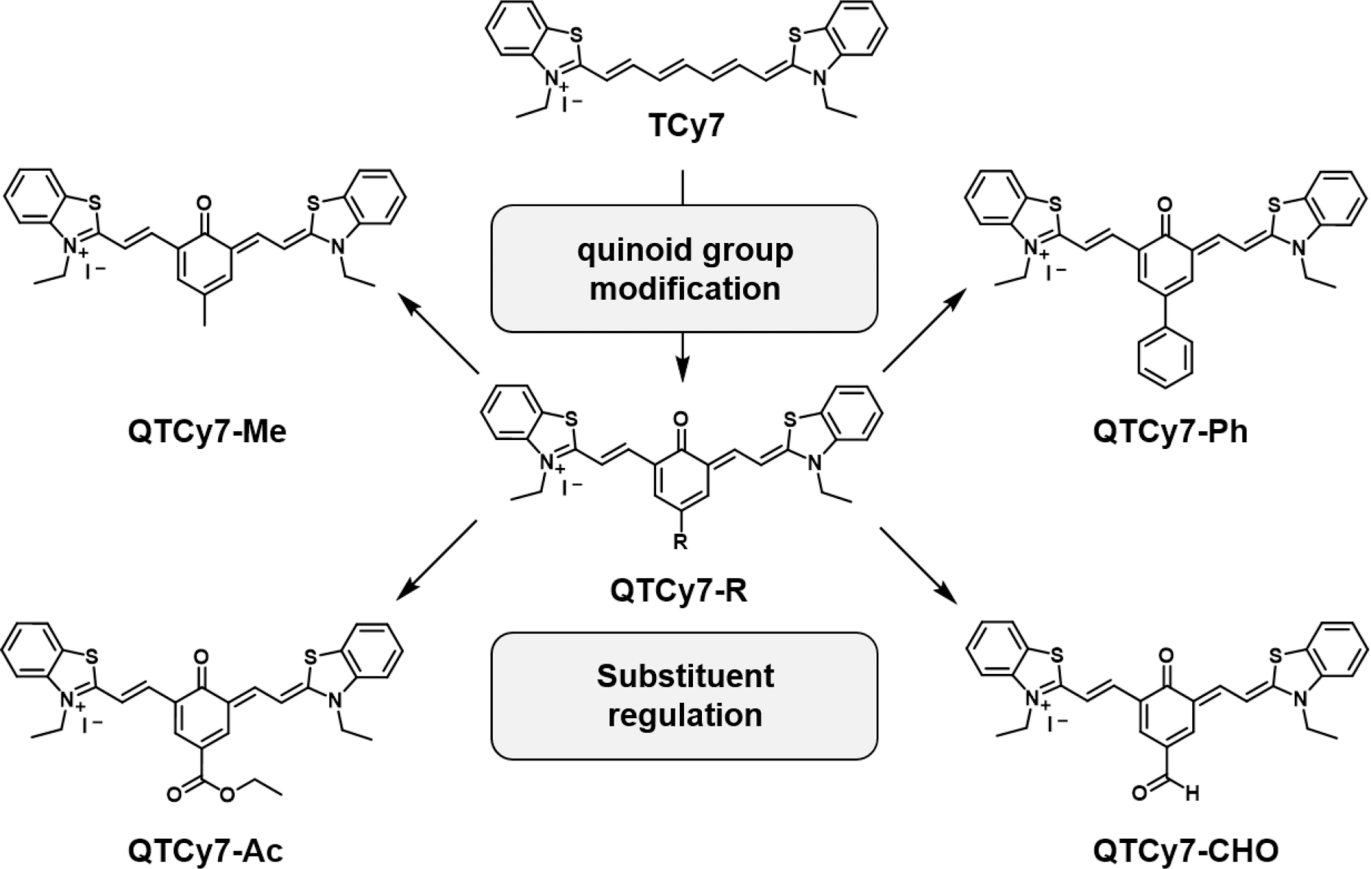
Chemical structures of TCy7 and QTCy7-R.

As a particular class of QCy dyes, QTCy7 compounds possess a long absorption and emission wavelength and adequate molar extinction coefficient in the NIR region, which benefits from the ^1^ππ* transition process of its excited state. By embedding a quinone structure in the conjugated chain of the molecule, the nonbonding orbital in the oxygen atom can participate in the process of excited-state electron transfer, resulting in the valid doping of the ^3^nπ* component in the low-lying triplet excited states, thus enhancing SOC from the ^1^ππ*–^3^nπ* mechanism.^39−41^ Simultaneously, the introduced rigid benzene ring structure also prevents *cis*–*trans* isomerization and rotation of the flexible chain, which partly reduce the thermal relaxation of the excited-state energy (Figure 1).

For QTCy7-R, all of the compounds were synthesized according to the synthetic routes detailed in Scheme S1. In brief, hydroxyphenylaldehydes containing different substituents (−Me, −Ph, −Ac, −CHO) were prepared by Duff reaction of the corresponding phenols with urotropine, and then the benzothiazole salt was reacted with the above condensation agents via Knoevenagel condensations. All the reaction products were fully characterized by ESI-HRMS, ^1^H NMR, and ^13^C NMR (Figures S24–S43).

### Determination of Spectra and Interpretation of Computational Mechanism

**Figure 2:**
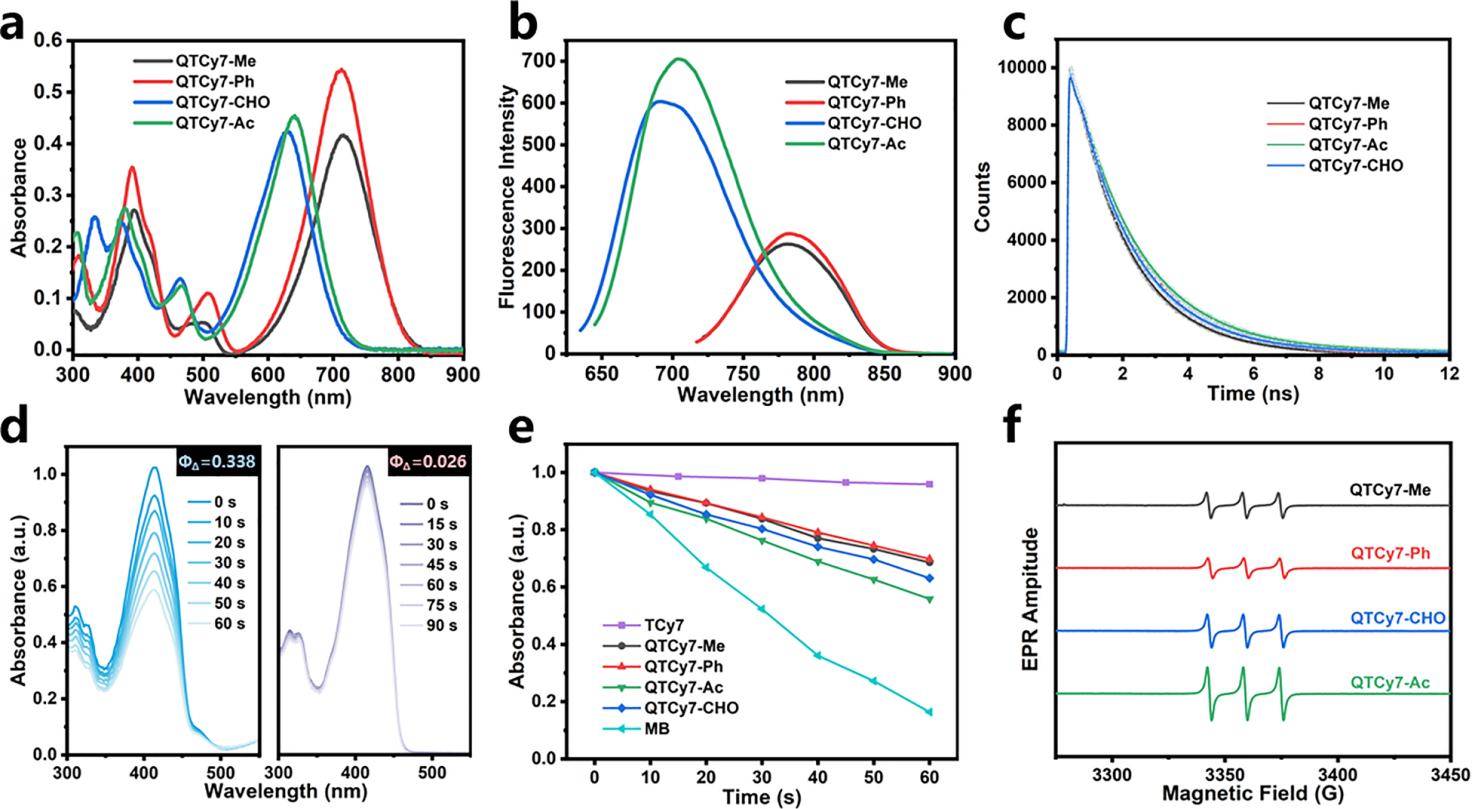
Spectral tests of QTCy7-R. (a) UV–vis absorption spectra and (b) fluorescence emission spectra of QTCy7-R (5 μM) in DCM. (c) Time-correlated single-photon counting fluorescence intensity decay of QTCy7-R. (d) DPBF degradation induced by QTCy7-Ac (left) and TCy7 (right) under 660 nm irradiation (2 mW/cm^2^). (e) Normalized DPBF degradation (415 nm) caused by different compounds under 660 nm irradiation in DCM. (f) EPR signals of ^1^O_2_ induced by different compounds under 660 nm irradiation in DCM.

First, the UV–vis absorption and fluorescence emission spectra of the series of compounds were measured and analyzed. QTCy7-Me and QTCy7-Ph displayed intense UV–vis absorption peaks at about 710 nm with the molar extinction coefficient of approximately 4 × 10^4^, while QTCy7-Ac and QTCy7-CHO exhibited a similar absorbance band at around 630 nm (Figure 2a), which indicated that the introduction of an electron-withdrawing group (EWG) could blue-shift the wavelength, and similarly, this blue-shift effect was also present in the emission spectra (Figure 2b). Instead, as shown in Table 1, conjugated EWG enhanced the fluorescence quantum yield (up to 28–31%), suggesting a strong NIR fluorescence imaging capability. Concurrently, the Stokes shifts of all four compounds were more than 60 nm (Figure 2a,b and Table 1), which could effectively improve the signal-to-noise ratio (SNR) of fluorescence compared to the ordinary TCy7. The fluorescence lifetimes of all four compounds were within 2 ns, indicating no delayed fluorescence generation (Figure 2c and Table 1). The UV–vis absorption spectra of the series compounds solvents of different polarity showed a small distinction, indicating a feeble solvation effect, which ensured the stability in different environments (Figure S1).

**Table 1:** Photophysical Properties of QTCy7-R

compd	λ_abs_ (nm)^a^	λ_em_ (nm)^b^	Δλ (nm)^c^	Φ_F_ (%)^d^	τ_S_ (ns)^e^	Φ_Δ_^f^
QTCy7-Me	714	780	66	16.7	1.70	0.225
QTCy7-Ph	711	783	72	17.2	1.90	0.212
QTCy7-CHO	630	690	60	28.0	1.82	0.263
QTCy7-Ac	636	703	67	31.4	1.97	0.338
TCy7	770	793	23	19.3	1.54	0.026

a Maximum absorption wavelength of the compound in dichloromethane.

b Maximum emission wavelength of the compound in dichloromethane.

c Stokes shift of QTCy7-R.

d Absolute fluorescence quantum yield.

e Fluorescence lifetime of QTCy7-R in dichloromethane.

f Singlet oxygen quantum yield in dichloromethane with methylene blue (MB) as the standard (Φ_Δ_ = 0.57 in dichloromethane).

Afterward, the singlet oxygen yields of the series of molecules were preliminarily measured and analyzed by the 1,3-diphenylisobenzofuran (DPBF) decay curve method (Figures 2d,e and S2). Compared with the reference TCy7, all QTCy7-R showed spectacular singlet oxygen generation ability (Table 1), particularly for QTCy7-Ac, which possessed much higher Φ_Δ_ than TCy7 (33.8% vs 2.6%). Meanwhile, in order to exclude the effect of the rigid structure, Φ_Δ_ of a Cy7-like reference molecule (TCy7C) with a rigidified structure but absence of an oxygen atom was measured. The singlet oxygen yield of TCy7C was 2.2%, even lower than that of TCy7 under the same conditions (Figure S3). Furthermore, the enhanced characteristic singlet oxygen signal (with TEMP as the scavenger) in electron paramagnetic resonance (EPR) measurements also showed similar results (Figure 2f). On the basis of this experimental evidence, we hypothesized that introduction of the quinone structure could be proved to effectively change the excited-state properties of TCy7 and improve the ISC process. Notably, the oxygen atom of the quinone structure preferred the n−π* transition in some cases, which therefore boosted the spin-forbidden electronic transition of singlet to triplet excited states through the ISC process to generate triplet excitons. Hence, the SOC enhancement caused by the ^1^ππ*–^3^nπ* process may be the emphasis of the ISC process.

**Figure 3:**
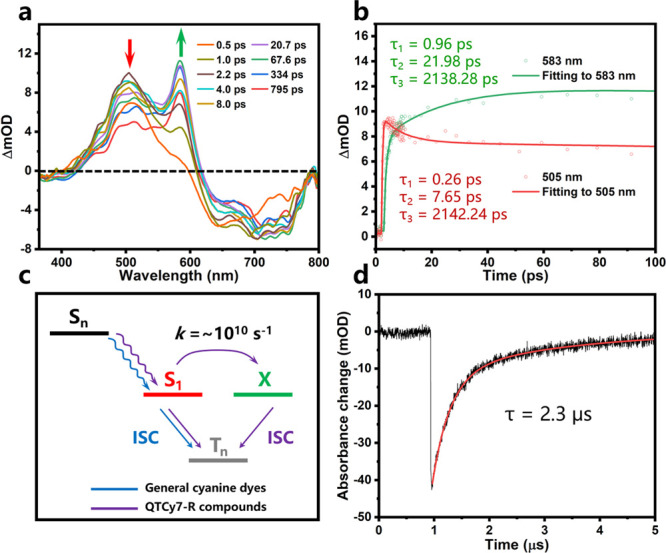
(a) Femtosecond transient absorption spectroscopy (fs-TA) analysis for QTCy7-Ac at different pump–probe delay times. Different color lines represent spectra at different times; λ_ex_ = 350 nm. (b) Kinetic traces and fitting lines of QTCy7-Ac taken through the representative ESA wavelength. (c) Schematic diagram of intersystem crossing process of excited states in general cyanine dyes and QTCy7-R compounds. (d) Kinetic traces of the triplet state of QTCy7-Ac (5 μM) in deaerated dichloromethane at 635 nm. λ_ex_ = 610 nm.

To probe the mechanism of the observed phenomenon and further validate our hypothesis, ultrafast excited-state dynamic behaviors were implemented by using femtosecond transient absorption spectroscopy (fs-TA) (Figures 3a and S4a–c). In order to obtain the complete process of excited states, a 350 nm laser was chosen as a pump to populate the high singlet excited states (S_*n*_) from the ground state (S_0_). Taking QTCy7-Ac as an example, according to the steady-state absorption spectra, it displayed distinct negative absorption bands at its maximum absorbance, which were assigned to the ground-state bleaching (GSB). As shown in Figure 3a,b, with a period of 260 fs internal conversion (IC) (S_*n*_–S_1_), QTCy7-Ac demonstrated a significant fast-rising positive absorption peak at ca. 505 nm, which was attributed to the excited-state absorption (ESA) of S_1_. With the growth of delay time, the ESA of S_1_ gradually decayed (τ = 7.65 ps); meanwhile, beyond our expectation, accompanied by a particularly noticeable peak at ca. 583 nm increasing promptly, this new emerging ESA peak reached its maximum within about 21.98 ps. Similar phenomena were found in the transient spectra of the other three compounds (Figures S4d–f). However, for the control TCy7, there was no obvious formation of another ESA peak in transient spectra (Figure S5). These peaks in QTCy7 were not simply ascribed to the generation of a triplet state, because its (hereinafter referred to as the X state) lifetime was similar to that of S_1_ (τ_X_ = 2.138 ns for this state and τ_S_1__ = 2.142 ns for S_1_). Furthermore, the rate of X state formation was calculated to be 4.54 × 10^10^ s^–1^ for QTCy7-Ac based on the rise time (*k*_X_ = 1/τ_X,rise_), which was close to the order of magnitude of the intramolecular distorted vibration behavior. Therefore, it was reasonable to assume that this process was controlled by the distorted vibration of the excited molecules. Moreover, such phenomenon in fs-TA of general cyanine photosensitizers with mediocre singlet oxygen yields was not observed in the earlier literature,^35^ indicating that the formation of the X state plays an important role in the ISC process (Figure 3c). Meanwhile, the triplet lifetime of this series of molecules was evaluated and inspected for further applications (Figures 3d and S4g–i). QTCy7-R exhibited almost reasonable triplet lifetimes, in which QTCy7-Me and QTCy7-Ph owned relatively long triplet lifetimes (about 12 μs) compared with QTCy7-Ac and QTCy7-CHO (about 2 μs). These lifetimes are basically enough for practical applications compared to the picosecond-level sensitization process between oxygen and molecules in excited states. In addition, due to the extremely weak triplet generation efficiency, the triplet lifetime of TCy7 was not obtained.

**Figure 4:**
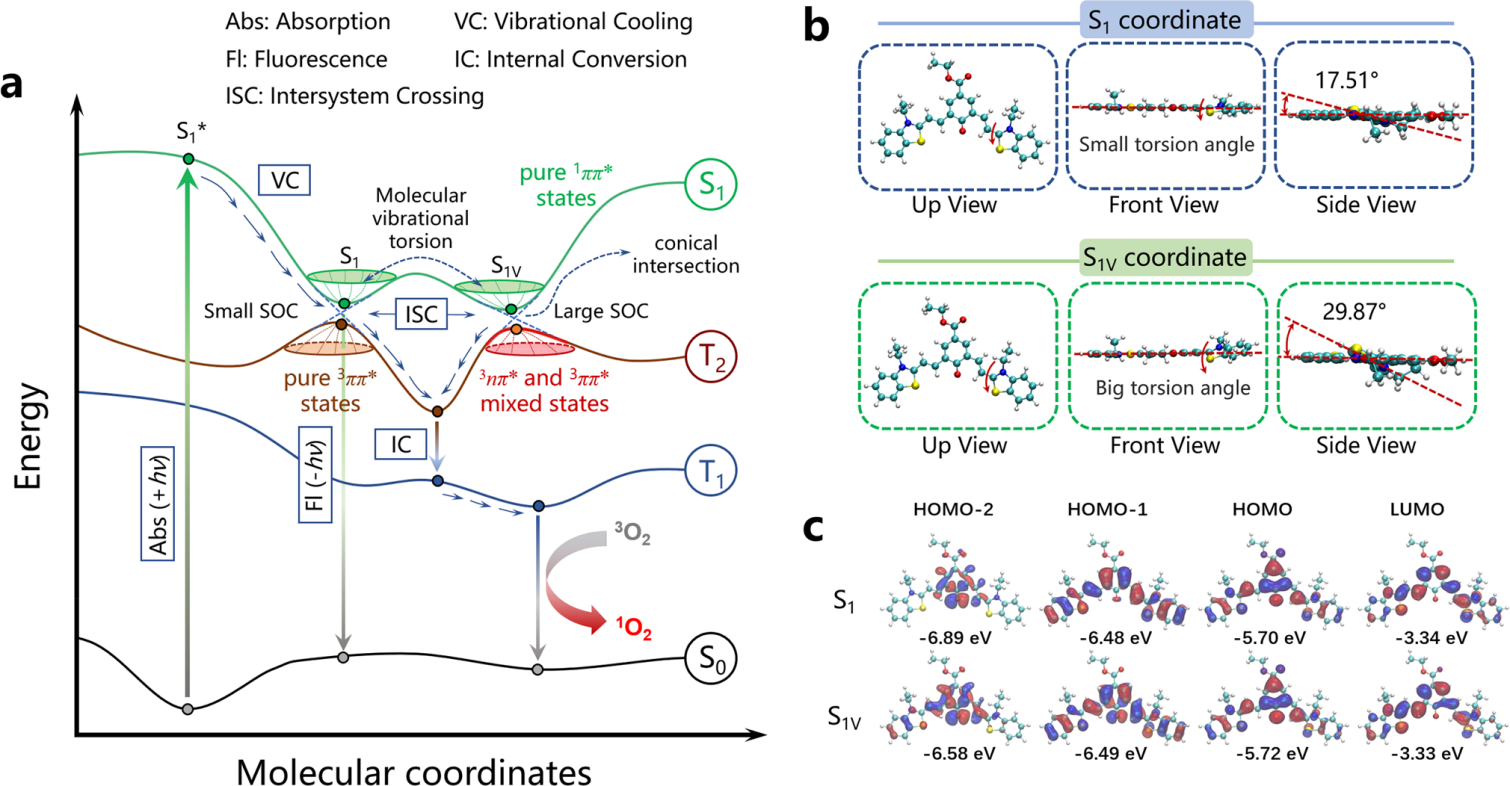
(a) Schematic diagram of the PVT-SOC mechanism in QTCy7-R. (b) Views from different locations (up, front, and side) of QTCy7-Ac and the torsion angles in the S_1_ and S_1V_ coordinates. (c) Frontier molecular orbitals (FMOs) and the corresponding energies of QTCy7-Ac in the S_1_ and S_1V_ coordinates.

In general, the S_1_–T_1_ transition can occur in two ways: (1) S_1_ is directly spin–orbit-coupled to the higher vibrational energy level of T_1_; (2) S_1_ is spin–orbit-coupled to T_*n*_, after which internal conversion from T_*n*_ to T_1_ proceeds. For method (1), the ISC rate depends on the energy gap between S_1_ and T_1_; however, for method (2), in addition to the energy gap between S_1_ and T_*n*_, we also expect that S_1_ should have a necessary vibrational motion to allow the molecule to look for an effective spin–orbit coupling mechanism in different conformations. Therefore, ISC is possible in all of the conformations of S_1_ isoenergetic points. Thus, the first-principles time-dependent density functional theory (TD-DFT) research on both singlet and triplet excited states was executed. According to the TD-DFT calculations, as depicted in Figure 4a, unlike ordinary molecules, compounds QTCy7-R had two steady energy-similar S_1_ states (Franck–Condon minimum), S_1_ and S_1V_, at almost the same energy (Δ*E* ⩽ 0.01 eV; Table S1). However, as shown for QTCy7-Ac in Figure 4b, these states had completely different molecular configurations, embodying the fact that configuration in S_1V_ had a larger out-of-plane torsion than S_1_, as its dihedral angle could reach about 30°. Simultaneously, the frontier molecular orbital (FMOs) at the S_1_ and S_1V_ coordinates were quite different (Figure 4c), with the HOMO–1 in the S_1V_ configuration exhibiting more n orbital characteristic on oxygen atom than the S_1_ configuration, while both S_1_ and S_1V_ had obvious n orbital characteristic in HOMO–2 and π/π* orbital characteristic in HOMO/LUMO, which provided a prerequisite for orbital transitions between excited states. A similar situation was observed in the other three QTCy7-R compounds (Figures S6 and S7a,b). On the contrary, there was no n orbital characteristic in the FMO of TCy7 (Figure S7c,d).

**Figure 5:**
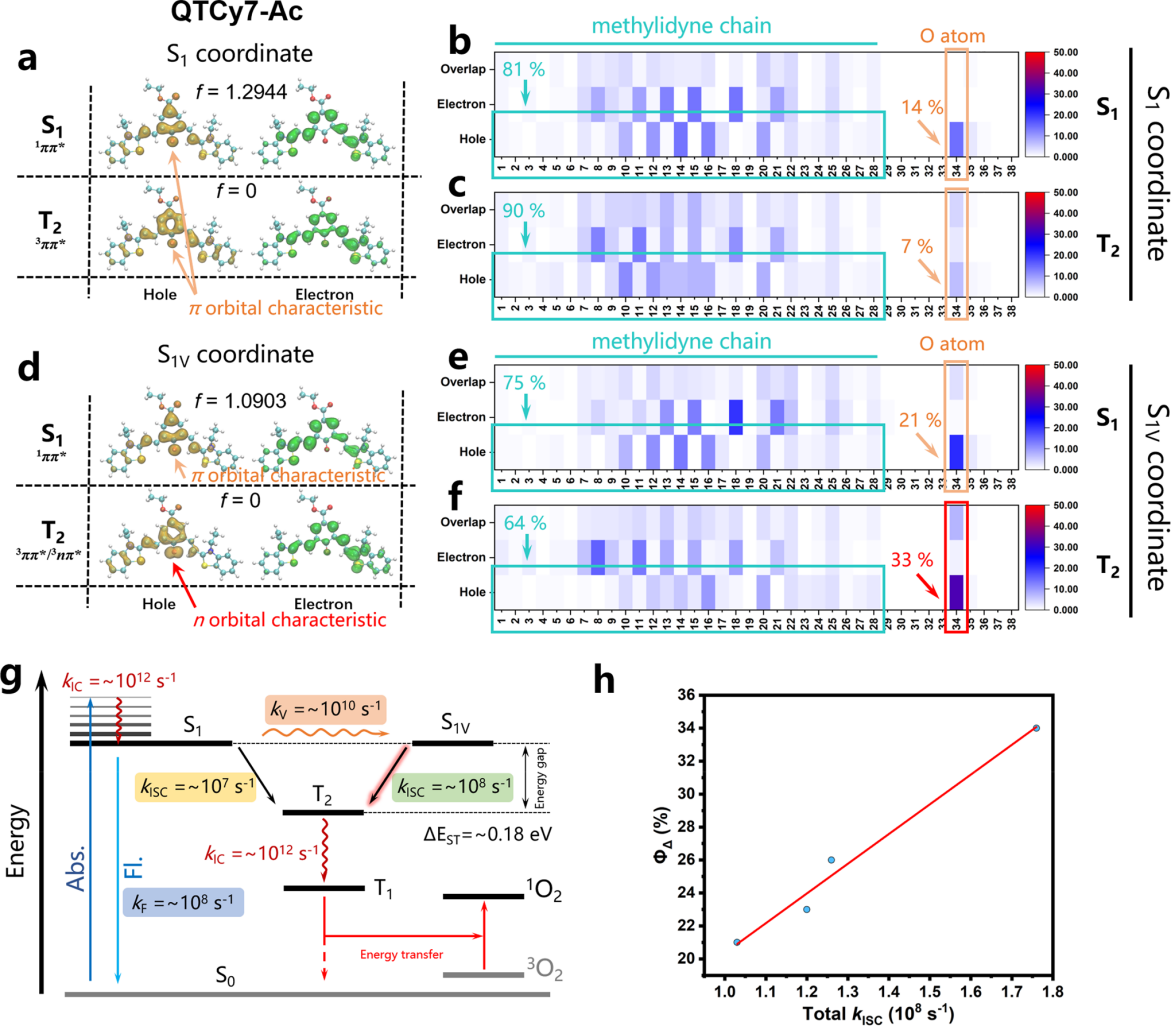
Hole–electron distribution and excited-state process analysis. (a) Hole–electron distribution of QTCy7-Ac at S_1_ and T_2_ in the S_1_ coordinate. (b, c) Heat maps of the hole–electron distributions of QTCy7-Ac at (b) S_1_ and (c) T_2_ in the S_1_ coordinate. (d) Hole–electron distribution of QTCy7-Ac at S_1_ and T_2_ in the S_1V_ coordinate. (e, f) Heat maps of the hole–electron distributions of QTCy7-Ac at (e) S_1_ and (f) in the S_1V_ coordinate. (g) Schematic diagram of different processes in the excited state of QTCy7-Ac and the orders of magnitude of their rate constants (*k*). (h) Positive correlation between Φ_Δ_ and total *k*_ISC_.

In order to describe the process of molecular excitation more clearly and intuitively, hole–electron analysis was utilized to investigate the characteristics of electron excitation, which was similar to natural transition orbital (NTO) analysis but more universal (all molecular orbital and hole–electron analyses were carried out using Multiwfn 3.8^42,43^). The first singlet excited state of all four compounds (QTCy7-R) was a bright locally excited (LE) state with typical π–π* characteristic whether it was in the S_1_ or S_1V_ configuration, as displayed for QTCy7-Ac in Figure 5a,d. As shown in the hole–electron distribution and heat map (Figure 5b,e; the atomic serial numbers are shown in Figure S8), the hole was concentrated on π orbitals of Q-carbonyl (ca. 14–21%) and the methylidyne chain (ca. 86–79%), while the electron was almost completely assembled at the methylidyne chain of QTCy7-Ac, indicating a large oscillator strength (*f* ≈ 1.2), which convincingly proved the formation of the LE singlet excited state with π–π* characteristic.

Pursuant to the energy gap law, the ISC process tends to take place at singlet and triplet states with close energy levels. As discovered in TD-DFT calculations, the second triplet state (T_2_) possessed the smallest energy gap to the S_1_ or S_1V_ state (Δ*E*_S_1_–T_2__ < 0.2 eV), which ensured efficient ISC premise. Furthermore, as depicted in hole–electron and heat map analysis (Figure 5a,c), the T_2_ state at the S_1_ configuration exhibited a significant π–π* characteristic, whose hole and electron were both restricted onto the π and π* orbitals of the methylidyne chain with tiny minority holes distributed around the n orbital of the oxygen atom. In this case, the SOC value between the S_1_(^1^ππ*) and T_2_(^3^ππ*) states would be relatively small, which was not conducive to the occurrence of ISC. Conversely, the T_2_ state at the S_1V_ configuration preferred to display n−π* excitation characteristic, in which the hole at the oxygen atom displayed a distinct dumbbell-like shape hovering on either side of the atom (Figure 5d). The formation of the ^3^nπ* state was mainly ascribed to the excitation between the n orbital part (33%) of the oxygen atom in the hole distribution and the π* orbital part of the methylidyne chain in the electron distribution. Relatively, the T_2_ state at the S_1_ configuration did not have any obvious n orbital characteristics, and the hole concentrated on the oxygen atom (7%) was also distinctly less than that under the S_1V_ configuration (Figure 5c,f). In other words, the T_2_ state at the S_1V_ configuration tended to possess more charge transfer (CT) state characteristic than the S_1_ configuration, which promoted the SOC by constructing the transition of ^1^LE → ^3^CT. On the contrary, the ^1^LE → ^3^LE transition would reduce the SOC on account of the ^1^ππ–^3^ππ* transition in the S_1_ configuration. Similarly, this feature was also reflected in the three other compounds (QTCy7-R) with similar structures (Figures S9–S11). Nonetheless, no analogous properties were found in TCy7 due to the absence of the S_1V_ coordinate (Figure S12).

In order to obtain an accurate evaluation of ISC theoretically, the ISC rate constants were calculated according to Marcus theory.^44,45^ According to the calculations, compared with the configuration at S_1_, the SOC values of S_1V_/T_2_ were significantly large due to the conservation of spin orbital angular momentum in the ^1^ππ*–^3^nπ* transition. Meanwhile, as described in Figure 5g and Table S2, the ISC rate constants (*k*_ISC_) in QTCy7-R at the S_1V_ configuration were an order of magnitude higher than those at the S_1_ configuration (∼10^8^ s^–1^vs ∼10^7^ s^–1^), which is large enough to compete with the fluorescence rate constant (*k*_F_ ∼ 10^8^ s^–1^). In sharp contrast, *k*_ISC_ in TCy7 of S_1_/T_2_ was extremely small (∼10^4^ s^–1^), which indirectly leads to a relatively small Φ_Δ_. In development, the total ISC rate constants had an obvious positive correlation with Φ_Δ_, which proved that the enhancement of ISC was a necessary condition for the improvement of Φ_Δ_ (Figure 5h).

**Figure 6:**
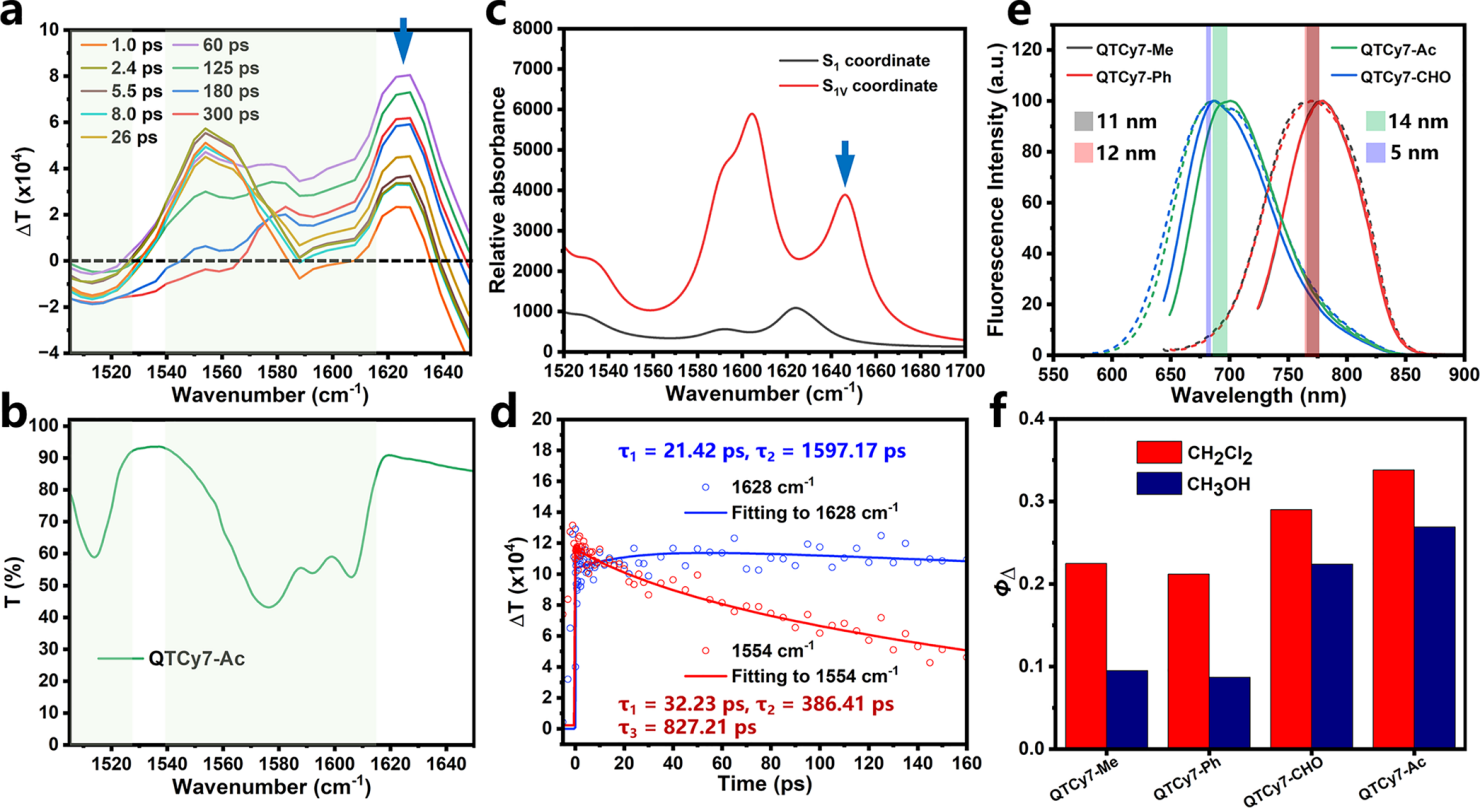
TRIR experiments and the comparison of excited-state properties between the DCM and MeOH of QTCy7-R. (a) TRIR spectra for QTCy7-Ac at different pump–probe delay times. Different colored lines represent spectra at different times. (b) FTIR spectrum for QTCy7-Ac.(c) Simulated IR spectra for QTCy7-Ac at the S_1_ and S_1V_ coordinates. (d) Kinetic traces and fitting lines of QTCy7-Ac taken through 1554 and 1628 cm^–1^. (e) Comparison of normalized emission spectra of QTCy7-R in dichloromethane and methanol. Solid lines represent spectra in dichloromethane, and dashed lines represent spectra in methanol. (f) Comparison of Φ_Δ_ of QTCy7-R in methanol and dichloromethane.

To further prove this conclusion, time-resolved infrared (TRIR) spectroscopy was conducted to study the vibrational modes of molecules at different coordinates in their excited states. Taking QTCy7-Ac as an example, the TRIR spectra possessed a superposed band containing both bleach vibrational bands and transient absorption bands at the wavenumbers from ca. 1505 cm^–1^ to ca. 1615 cm^–1^ (Figure 6a,b). With a longer time, a new prominent absorption band at ca. 1628 cm^–1^ appeared and gradually enhanced (Figure 6a, blue arrow), which indicated that a new vibrational pattern was generated in excited states. Therefore, the vibrational modes of QTCy7 in the S_1_ and S_1V_ coordinates were calculated, and the results were plotted as simulated IR spectra. As depicted in Figure 6c, compared with the spectra at the S_1_ coordinate, a new absorption band at ca. 1644 cm^–1^ was observed only at the S_1V_ coordinate, which possessed high similarity with the results measured in the TRIR experiment. Furthermore, the vibrational vector at this absorption band was analyzed (Figure S16), and the vibrational mode was ascribed to stretching and oscillating vibration of the methylidyne chain (It is worth noting that the obvious absorption band at ca. 1623 cm^–1^ in Figure 6b was ascribed to vibration of the benzene ring in benzothiazole group, which appeared in either the S_1_ coordinate or S_1v_ coordinate.). Simultaneously, exponential fitting also indicates that the vibrational mode at ca. 1628 cm^–1^ tended to be at a maximum at about 22.65 ps (Figure 6d), which was on the same order of magnitude as the data obtained in the fs-TA experiments (21.98 ps). Analogously, similar phenomena had been found in TRIR experiments of QTCy7-Me and QTCy7-Ph (Figures S13, S14, and S16). Compared with QTCy7, the control TCy7 only showed bleach vibrational bands at the wavenumbers of ca. 1539 cm^–1^, but no similar transient absorption bands were observed (Figure S15). However, due to the poor photostability of QTCy7-CHO under the experimental conditions, effective data were not obtained. Hence, it is reasonable to suppose that the changes in TRIR spectra reflect the molecular coordinate change (S_1_–S_1V_) by PVT in excited states.

Meanwhile, as a proof of principle, the fluorescence emission spectra and Φ_Δ_ in DCM and MeOH of all four compounds were evaluated and analyzed to illustrate the forecast. The fluorescence emission spectra in DCM and MeOH exhibited similar wavelengths (Δλ < 15 nm), which implied that the energy of the ^1^ππ* state was scarcely influenced (Figure 6e). On the other hand, it was widely accepted that formation of a hydrogen bond between the carbonyl group and protic solvent would lead to instability of the ^3^nπ* state and therefore increase its energy,^46,47^ which further reduced the efficiency of the ^1^ππ*–^3^nπ* transition and ISC process. As we expected, the Φ_Δ_ in MeOH demonstrated an apparent quenching compared with that in DCM (Figures 6f and S17), which supplied a strong piece of circumstantial evidence for the hypothesis that the formation of the ^3^nπ* state was important in the S_1V_ → T_2_ transition process.

From what has been discussed above, the formation of X states from S_1_ in fs-TA can be attributed to a high probability of the S_1_ → S_1V_ conversion process. As a matter of fact, the intramolecular vibrational torsion indeed occurs on approximately the picosecond time scale, which provides a credible precondition for the PVT-SOC mechanism to be proposed. In our theory, there are three necessary prerequisites for the PVT-SOC mechanism: (1) there must be an intramolecular vibrational torsion motion in the excited states which is faster than the emission of the fluorescence process; (2) the vibrational torsion motion must bring the molecule to an energy-close thermodynamic steady-state point (Franck–Condon minimum) at the potential energy surface; (3) the mixing of electronic states caused by the distortion vibration must inevitably lead to the increase of SOC at this Franck–Condon point/conical intersection point (schematic diagram of PVT-SOC in Figure 4a).

### *In Vitro* Application

In order to further investigate the PDT ability of QTCy7-R on cancer cells, the UV–vis absorption and emission spectra and ^1^O_2_ production capacity of QTCy7-R were tested in phosphate-buffered solution (PBS) to better mimic the cellular environment. As shown in Figure S18, compared with UV–vis absorption spectra in organic solution, the spectra of QTCy7-R in PBS all have different degrees of blue shift. QTCy7-Me (701 nm) and QTCy7-Ac (610 nm) were relatively less affected, while QTCy7-Ph (613 nm) and QTCy7-CHO (552 nm) showed distinct blue shifts, which might be due to the aggregation of them in aqueous medium. Meanwhile, the fluorescence quenching of QTCy7-Ph and QTCy7-CHO also confirmed their aggregation in aqueous solution compared to the intense fluorescence emission of QTCy7-Ac. Furthermore, singlet oxygen sensor green (SOSG) was selected to investigate the ^1^O_2_ quantum yield in PBS. As depicted in Figure S18i, compared with the control TCy7, all QTCy7-R exhibited considerable ^1^O_2_ production capacity, especially QTCy7-Ac, whose ^1^O_2_ quantum yield was an order of magnitude bigger than those of the other QTCy7 and even 5.8 times as much as that of MB.

In view of QTCy7-R having strong singlet oxygen generation potency and reasonable wavelength, human hepatocellular carcinoma cells were selected for photoactivity experiments *in vitro*. On account of their low molecular weights and intrinsic positive cationic charge, QTCy7-R could be quickly taken up by cells within 90 min (Figure S19a,b). Therein, QTCy7-Me and QTCy7-Ph could get inside cells due to the evident fluorescence inside the cells. In contrast, it could be sufficiently indicated that QTCy7-Ac or QTCy7-CHO could effectively adhere to the cell membrane by the obvious red fluorescence signal on the cell edge. According to our supposition, the enhanced partial cationic charge caused by the introduction of the EWG in QTCy7-Ac or QTCy7-CHO made it easier for them to cling to the cell membrane with negative charge than to cross it.

**Figure 7:**
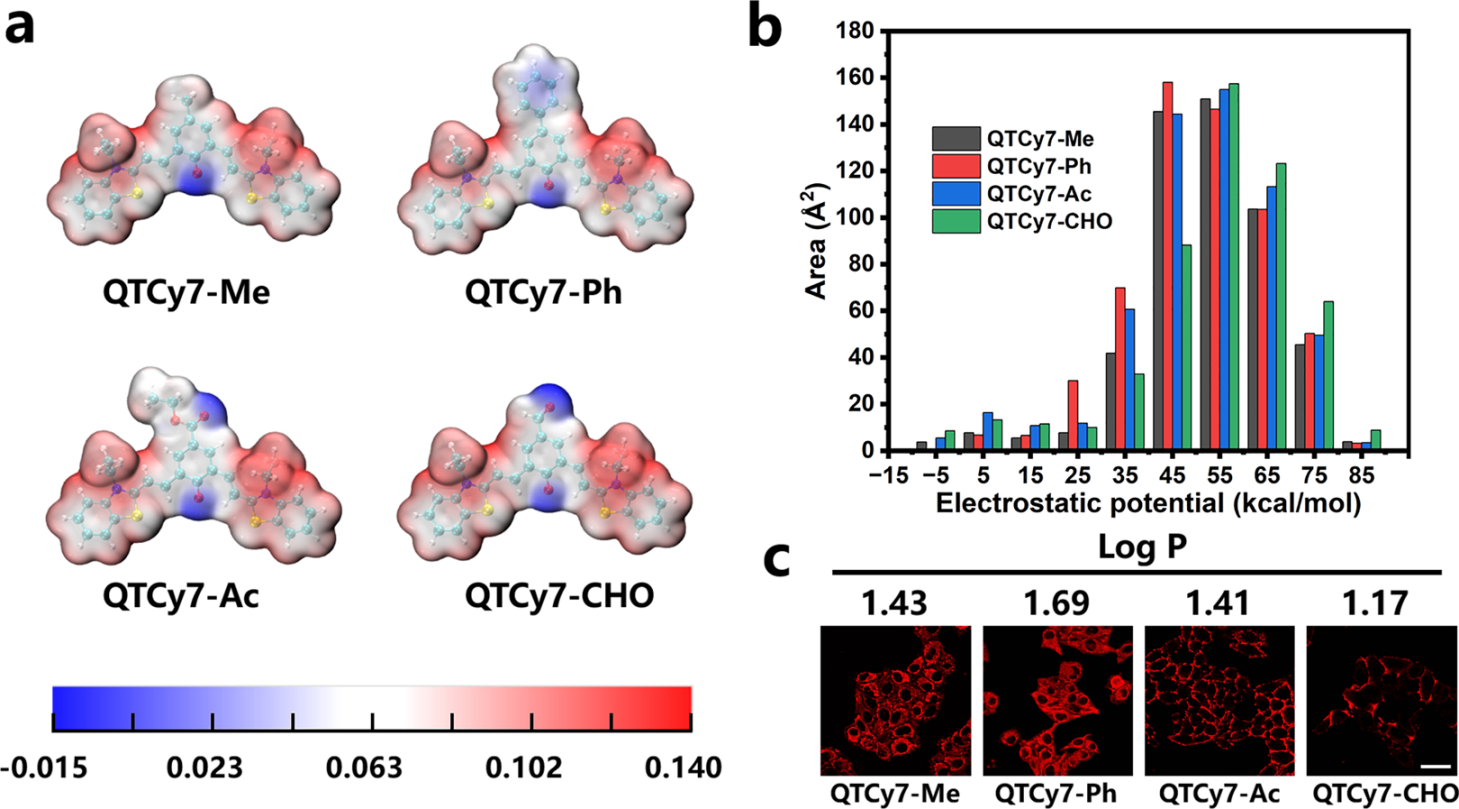
(a) Electrostatic potential analysis of QTCy7-R. (b) Area distribution of different ESP intervals in different compounds. (c) Log P and cellular uptake of QTCy7-R in HepG2 cells after being incubated for 90 min (scale bar: 20 μm).

In order to guarantee the preciseness of the supposition, electrostatic potential (ESP) analysis of the molecular surface was conducted to quantify the surface charge distribution.^48^ With a unit positive charge, the surface potential of the molecule was mostly positive. As depicted in Figure 7a, most of the positive electrostatic potential regions on the surface of the molecules were located on the benzothiazole groups in all four compounds, while only a small amount of positive electrostatic potential and negative electrostatic potential are distributed on the benzoquinone group and the oxygen atom, respectively. Furthermore, in the high surface positive potential region (>55 kcal/mol), QTCy7-CHO possessed the largest effective surface area, which was ascribed to the strong electron withdrawing properties of the aldehyde group. On the contrary, QTCy7-Ph preferred a larger effective surface area at the low surface positive potential region (<55 kcal/mol). In addition, the areas of surface electrostatic potential in QTCy7-Me and QTCy7-Ac were maintained in a median (QTCy7-Ac was partly larger than QTCy7-Me) (Figure 7b). The oil–water partition coefficient (Log P) was also taken into account to study cell uptake. According to Log P (Figure 7c), the introduction of phenyl enhanced the lipophilicity of QTCy7-Ph, resulting a good membrane permeability, whereas the introduction of formyl enhanced the hydrophilicity of QTCy7-CHO, which decreased its cellular uptake capacity. For QTCy7-Me and QTCy7-Ac, although they had a similar Log P, QTCy7-Me was better than QTCy7-Ac in cell membrane permeability due to the influence of the surface potential distribution. Interestingly, the distribution of electrostatic potential on the surface and Log P of molecules was obviously correlated with the uptake of the four compounds: the stronger the positive electrostatic potential on the surface, the harder it is to cross the cell membrane, and the better the hydrophilicity, the harder the cellular uptake. This explains why only a small amount of QTCy7-CHO adhered on the membrane surface (Figure 7c).

**Figure 8:**
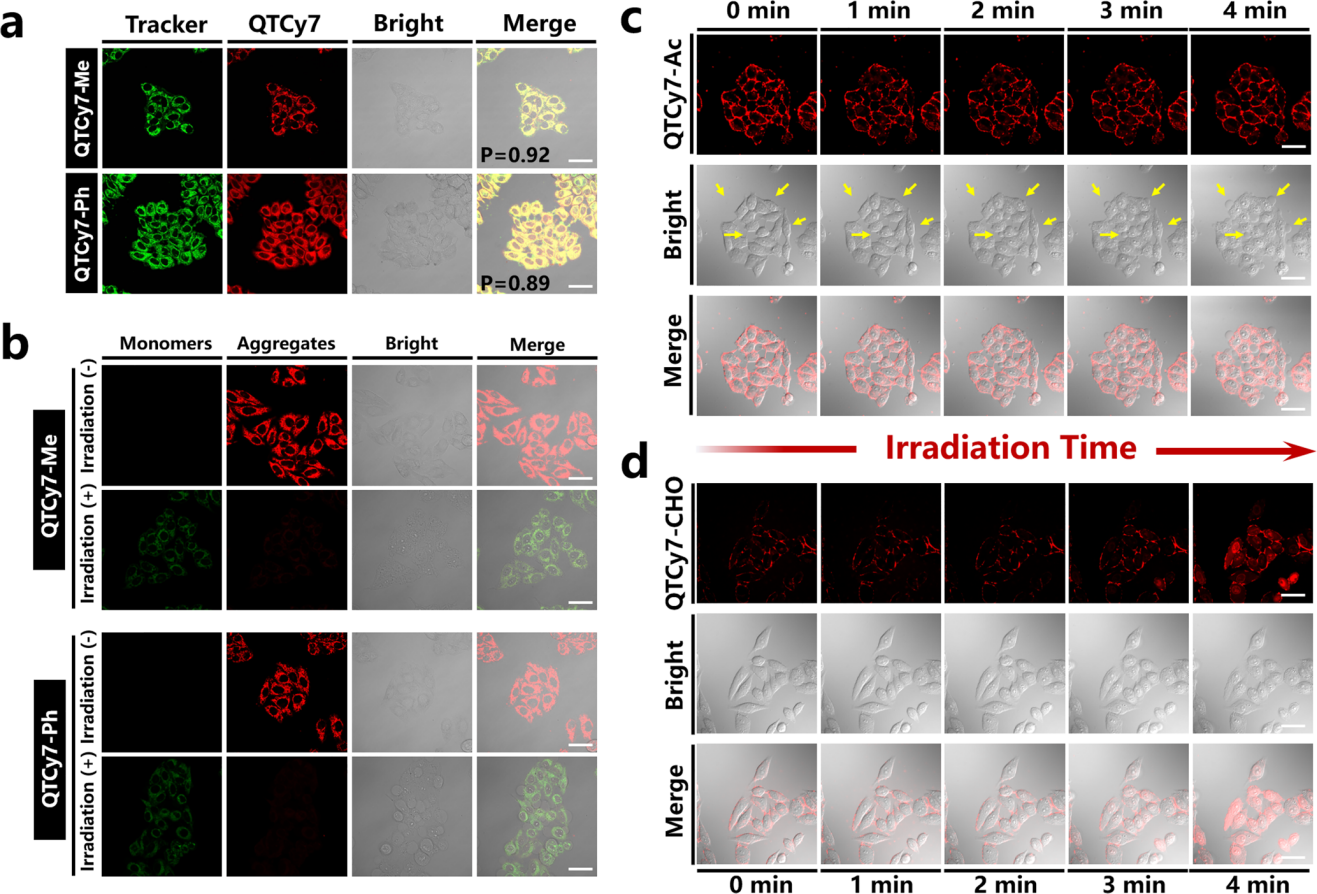
Confocal laser scanning microscopy (CLSM) imaging of cell uptake and damage. (a) Subcellular colocalization images of QTCy7-Me/QTCy7-Ph and MitoTracker Green (MTG) in HepG2 cells; *P* is the colocalization coefficient. (MTG: λ_ex_ = 488 nm, λ_em_ = 500–550 nm; QTCy7-Me/QTCy7-Ph: λ_ex_ = 640 nm, λ_em_ = 700–800 nm; scale bars: 20 μm). (b) Mitochondrial membrane potential detection assays implemented by JC-1 staining. (JC-1 monomer: λ_ex_ = 488 nm, λ_em_ = 500–550 nm; JC-1 aggregate: λ_ex_ = 488 nm, λ_em_ = 560–590 nm; scale bars: 20 μm). (c) QTCy7-Ac and (d) QTCy7-CHO caused cell membrane destruction images at 0–4 min (λ_ex_ = 640 nm; λ_em_ = 650–750 nm; scale bars: 20 μm).

Interestingly, QTCy7-Me and QTCy7-Ph had highly efficient accumulation in cell mitochondria, which could be verified by the intracellular distribution measurement using commercial subcellular organelle localization trackers (Figure 8a). The red fluorescence signal produced by QTCy7-Me or QTCy7-Ph overlapped well with the green fluorescence representing MitoTracker Green in mitochondria (Pearson correlation coefficient of 0.92 and 0.89 for QTCy7-Me and QTCy7-Ph, respectively), which proved that there is a high affinity between QTCy7-Me or QTCy7-Ph and mitochondria. Next, in order to verify the disruption effect on cell structural integrity of the ^1^O_2_ generation by QTCy7-R under irradiation, JC-1 staining imaging was conducted to monitor the mitochondria membrane potential. As depicted in Figure 8b, under 660 nm irradiation (6 J/cm^2^), QTCy7-Me and QTCy7-Ph could induce severe mitochondrial depolarization accompanied by the significant weakening of red fluorescence signal (JC-1 aggregates) and enhancement of the green fluorescence signal (JC-1 monomers). On the other hand, cell membrane damage caused by the photooxidation of QTCy7-Ac and QTCy7-CHO could be confirmed by a visualized observation. With the extension of illumination time, the formation of distinct bubblelike structures could be observed on the surface of the cell membrane after being incubated with QTCy7-Ac (Figures 8c and S20a), which proved the destruction of the integrity of the cell membrane and extravasation of intracellular material caused by ^1^O_2_. In contrast, QTCy7-CHO did not cause severe cell membrane rupture because of a slight change in cell morphology (Figures 8d and S20b). Nevertheless, unlike QTCy7-Ac, the fluorescence intensity of QTCy7-CHO gradually enhanced from the membrane surface to the interior of the cell, indicating the change of cell membrane permeability, which thus inferred that QTCy7-CHO could also destroy the cell structure under irradiation. Simultaneously, more importantly, the fluorescence intensity in cells did not weaken during light irradiation, proving the good photostability of QTCy7-Ac/QTCy7-CHO as an efficient photosensitizer. Furthermore, the lactate dehydrogenase (LDH) content in cell culture medium was measured to characterize the extent of cell membrane damage.^49^ As shown in Figure S19c, the LDH content exhibited a remarkable increase after incubation with QTCy7-Ac compared to QTCy7-CHO under irradiation, and its release rate reached about 70%, indicating a fierce cell membrane destruction capacity.

**Figure 9:**
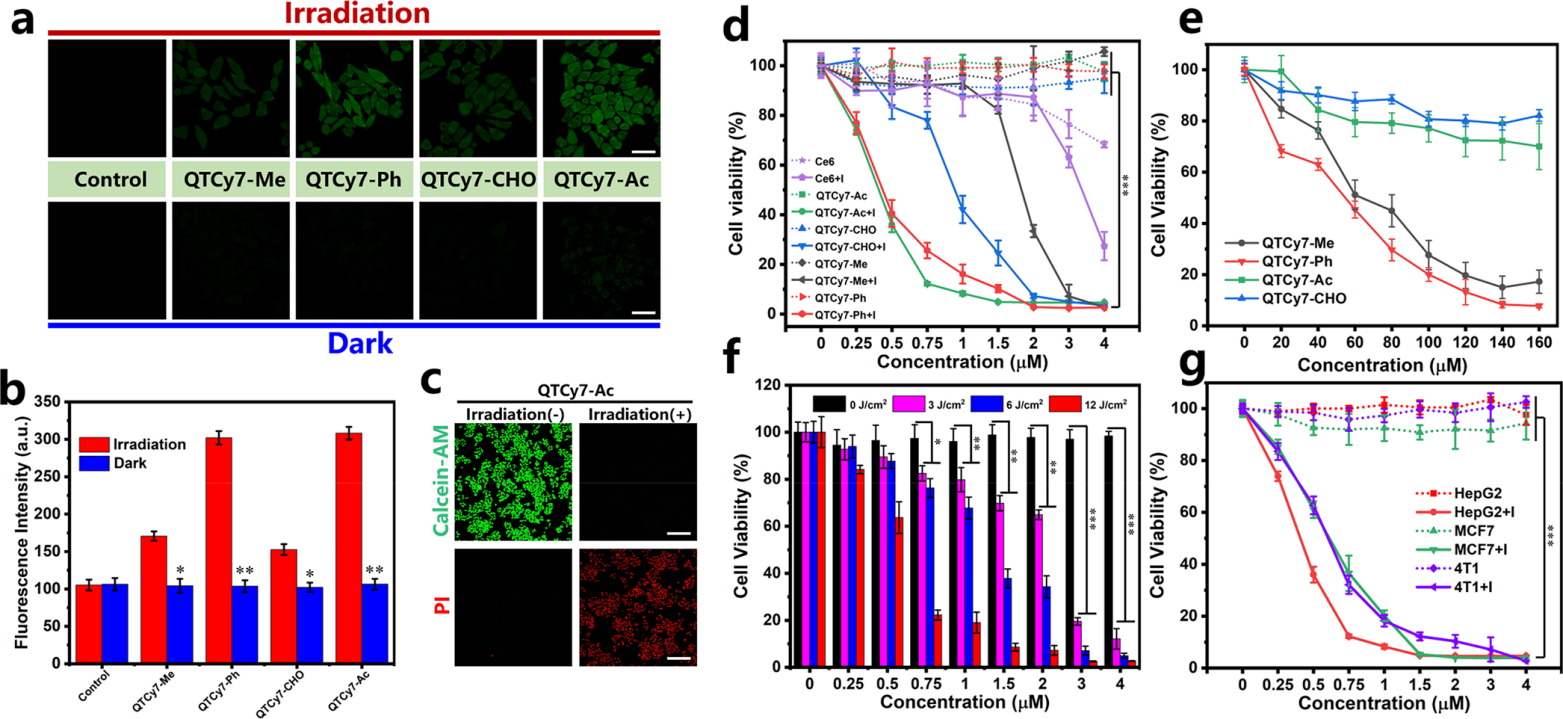
Intracellular ROS production and cell destruction assays. (a) Images showing intracellular ROS production induced by QTCy7-R under irradiation and dark conditions (λ_ex_ = 488 nm; λ_em_ = 500–550 nm; scale bars: 20 μm) and (b) corresponding fluorescence intensities. (c) Cell viability detection images measured by Calcein-AM/PI for QTCy7-Ac (Calcein-AM: λ_ex_ = 488 nm, λ_em_ = 500–550 nm; PI: λ_ex_ = 561 nm, λ_em_ = 580–630 nm; scale bars: 120 μm). (d) Quantitative detection of cell viability (MTT assay) for QTCy7-R and Ce6 with/without 660 nm irradiation (12 J/cm^2^). (e) Cell viability of HepG2 cells treated with QTCy7-R at different concentrations under dark condition. (f) Cell viability of HepG2 cells treated with QTCy7-Ac under different light doses. (g) Cell viability of different cancer cells (HepG2, MCF7, and 4T1) treated with QTCy7-Ac under 660 nm irradiation (12 J/cm^2^). Data are expressed as mean ± SD. **, *P* < 0.01; ***, *p* < 0.001; ****, *P* < 0.0001 as determined by Student’s *t* test.

In order to explore the mechanism of cellular structure destruction caused by QTCy7-R, intracellular ROS generation was investigated by DCFH-DA reagent. As exhibited in Figure 9a, there was an obvious fluorescence enhancement upon treatment with QTCy7-R in contrast to the control group after light irradiation, indicating that these four molecules could produce ROS in cells. Among them, QTCy7-Ph and QTCy7-Ac showed a stronger fluorescence signal (Figure 9b). Ulteriorly, cell destruction caused by ROS generation was assessed by Calcein-AM/propidium iodide (PI)-mediated fluorescence imaging (Figures 9c and S21). Compared with the intense green fluorescence signal in living cells, after illumination with 660 nm irradiation (10 mW/cm^2^) for 5 min, the strong red fluorescence signal generated by PI entering the nuclei of dead cells indicated that all four molecules can effectively kill cells.

To further quantitatively explore the *in vitro* anticancer potency of QTCy7-R, the methyl thiazolyltetrazolium (MTT) assay was employed to detect cell viability. As shown in Figure 9d, all four compounds exhibited negligible cytotoxicity toward HepG2 cells in dark condition, yet they could effectively inhibit cell proliferation in a concentration-dependent manner under the same irradiation condition. QTCy7-CHO and QTCy7-Me showed relatively general inhibition in cell growth, and their half-maximal inhibitory concentration (IC_50_) values were about 1.8 and 0.9 μM, respectively. In contrast, a powerful cell-killing capability was demonstrated for QTCy7-Ph and QTCy7-Ac with nearly the same IC_50_ of about 0.3 μM after exposure to NIR light. Notably, ROS production of these four molecules showed a positive correlation with the cell damage ability, illustrating that ^1^O_2_ generation was the direct cause of cell death. Moreover, the cell growth inhibition effect of all QTCy7-R was better than that of commercial chlorin e6 (Ce6) photosensitizer (IC_50_ = 3.5 μM), which demonstrated that this series of molecules possessed excellent cell killing capacity. Next, we evaluated the cytotoxicity under dark conditions of this series of compounds at high concentrations (Figure 9e), as due to the characteristics of mitochondrial accumulation, QTCy7-Me and QTCy7-Ph might lead to mitochondrial depolarization and apoptosis at high concentrations. However, its IC_50_ value could still reach about 60 μM, which was almost lower than those of all cyanine photosensitizers reported. On the other hand, 70–80% of cells could still survive even at an exceedingly high QTCy7-Ac or QTCy7-CHO concentration of around 160 μM, indicating that the intracellular microenvironment would not be disturbed owing to their adhesion properties on cell membranes. The photocytotoxicity index value (dark IC_50_/light IC_50_) of QTCy7-Ac was >500, manifesting that QTCy7-Ac is a PS with high efficacy but low cytotoxicity, which is extremely promising in clinical application.

Hence, the cell inhibition effect of QTCy7-Ac was further investigated under different light doses (0, 3, 6, and 12 J/cm^2^) (Figure 9f). The IC_50_ values were about 2.0, 1.2, and 0.3 μM in the light of 3, 6, and 12 J/cm^2^, respectively, which suggested a remarkable potency for QTCy7-Ac to generate ^1^O_2_ under weak light excitation. Moreover, to further prove the nonselective cell killing ability of QTCy7-Ac, two other cancer cells (murine mammary carcinoma cells (4T1) and human mammary carcinoma (MCF7)) were used to detect the growth inhibition potential. As shown in Figure 9g, the IC_50_ values for them were about 0.5 and 0.6 μM, indicating its broad-spectrum cancer cell destruction as a photosensitizer. These results clearly demonstrated that nascent QTCy7-Ac photosensitizer under the new ISC-enhanced strategy played a powerful role in the inhibition of cancer cells.

### *In Vivo* Application

**Figure 10:**
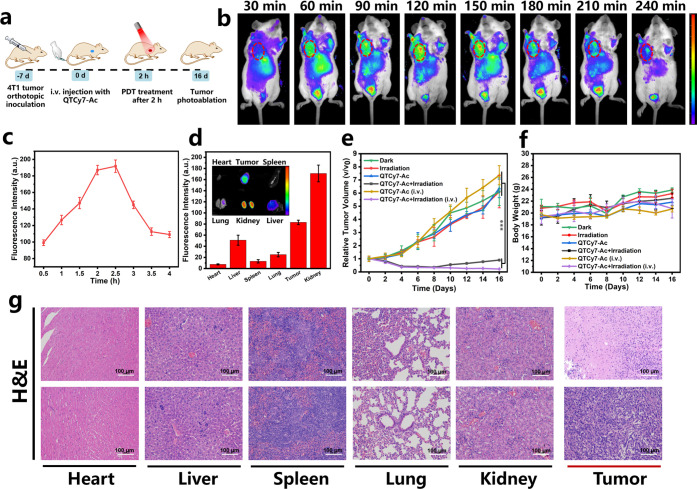
*In vivo* solid tumor imaging and inhibition tests. (a) Schematic diagram of PDT for solid tumors mediated by QTCy7-Ac. (b) *In vivo* real-time fluorescence imaging of 4T1 subcutaneous solid tumor mice after iv injection of QTCy7-Ac. (c) Relative fluorescence intensities at different times (λ_ex_ = 660 nm, λ_em_ = 720 ± 20 nm). (d) Fluorescence imaging and relative fluorescence intensities of main organs and tumor after iv injection of QTCy7-Ac for about 2.5 h (λ_ex_ = 660 nm, λ_em_ = 720 ± 20 nm). (e) Relative tumor volumes with different treatments at different days. (f) Body weights of the mice in various groups at different days. (g) H&E staining assays of main organs and tumors in the QTCy7-Ac + irradiation (i.v.) group (top) and QTCy7-Ac (i.v.) group (bottom) after 16 days of treatment (scale bars: 100 μm). Data are expressed as mean ± SD. **, *P* < 0.01; ***, *p* < 0.001; ****, *P* < 0.0001 as determined by Student’s *t* test.

The prospective *in vitro* results prompted us to further explore *in vivo* application possibility of QTCy7-Ac, and a solid tumor model of BALB/c mice was established by inoculating 4T1 cells subcutaneously under the armpit (Figure 10a). All the BALB/c mice were purchased from Liaoning Changsheng Biotechnology Co., Ltd., and the animal experiments were approved by the Local Scientific Research Ethics Review Committee of the Animal Ethics Committee of Dalian University of Technology (ID no. // Ethics Approval No. 2021-043). When the tumor volume reached 100 mm^3^, fluorescence imaging was performed at different time points after intravenous injection of QTCy7-Ac. As we expected, due to the inherent targeting of Cy7-type molecules in cancer cells,^50−52^ the tumor region was quickly “lit up” around 60 min (Figure 10b), and the fluorescence signal gradually increased, reaching the maximum value around 2.5 h (Figure 10c). Subsequently, the fluorescence intensity in the tumor site gradually decreased significantly within 4 h, indicating the rapid metabolism and good safety of QTCy7-Ac *in vivo*. Meanwhile, in the process of fluorescence imaging, an interesting phenomenon was that the bladder region of the mice also showed strong fluorescence signal, which we speculated was related to the dye metabolism through the kidney into the urine. To test this hypothesis, the mice were executed and dissected 2.5 h after intravenous injection, and the fluorescence imaging of the main organs and tumor was performed. As shown in Figure 10d, as we suspected, unlike most cyanine dye molecules that were metabolized through the liver,^24,25^ the kidney showed a convincingly strong fluorescence signal, suggesting that the dye was metabolized primarily through the kidney. Compared with liver metabolism, drugs metabolized by the kidney have been proved to have better biocompatibility,^53,54^ which further indicated that QTCy7-Ac has certain medical application value. Simultaneously, the tumor also showed a non-negligible fluorescence signal stronger than the liver, which demonstrated excellent enrichment in the tumor site.

In view of the observation that the fluorescence intensity of QTCy7-Ac in the tumor site peaked around 2–2.5 h after intravenous injection, this period was chosen as the PDT time window. After a single PDT treatment (660 nm, 100 mW/cm^2^, 15 min) (Figure 10e), the groups containing QTCy7-Ac by intratumoral injection initially exhibited ideal tumor inhibition, but the tumor volume increased a little after 8 days, which was caused by the treatment blind spot ascribed to uneven diffusion of intratumoral injection drugs. Even so, this approach achieved partial tumor suppression; the tumor volume was 1-fold compared with the initial situation. However, the groups containing QTCy7-Ac by intravenous injection exposed to NIR light showed extraordinary tumor regression. After PDT treatment, the tumor kept shrinking in size (Figure S22a), which achieved a tumor suppression rate of nearly 95% (Figure S22b). Nevertheless, the tumors in PBS groups increased quickly with a 6-fold increment compared with the initial volume whether exposed to irradiation or not, which ruled out the influence of light on tumor growth. In addition, there was virtually no difference between the intratumoral and intravenous injection groups treated with QTCy7-Ac, with tumor volumes increasing by 6-fold and 7-fold, respectively, which excluded the cytotoxicity of the dye alone.

The efficacy and safety of each therapeutic group were also evaluated by performing a hematoxylin and eosin (H&E) staining assay of major organs and tumor tissue (Figures 10g and S23). Conspicuous morphological damage was observed in tumor tissues treated with QTCy7-Ac-mediated PDT. The distinct cell necrosis and inflammatory response in any major organ sections including heart, liver, spleen, lung, and kidney were not observed, and no abnormal body weight changes were obtained in mice during the treatment (Figure 10f), which further underscored that QTCy7-Ac possessed good biocompatibility and applicability *in vivo*.

## Conclusion

In summary, a novel property called photoinduced molecular vibrational torsion (PVT)-enhanced spin–orbit coupling (PVT-SOC) to enhance intersystem crossing of QTCy7 photosensitizer was first revealed and applied for PDT successfully. By modifying QTCy7 with an ester group and utilizing intramolecular vibrational torsion motion, a series of NIR photosensitizers with high ^1^O_2_ yield (33.8%) and reasonable fluorescence quantum yield (31%) were successfully obtained. Combining computational theoretical calculation, transient absorption spectroscopy, and other experimental evidence, the enhanced ISC efficiency was clarified logically as the PVT-SOC mechanism: the photoinduced molecular vibrational torsion motion in the S_1_ state would bring the molecule to another energy-close Franck–Condon minimum (S_1V_) at the potential energy surface. Under this circumstance, the ^1^ππ*–^3^nπ* transition between S_1V_ and T_2_ greatly increased the SOC, which subsequently strengthened *k*_ISC_ (∼10^8^ s^–1^). QTCy7-Ac exhibited optimal photophysical properties and demonstrated excellent PDT potential owing to its low cytotoxicity and broad-spectrum cell membrane destruction at low light doses compared with commercial Ce6 photosensitizer (11-fold enhancement). The structural inherent targeting of tumors endowed QTCy7-Ac to possess a strong ability to accumulate in tumor sites and perform effective tumor photoablation (95%) under NIR irradiation. Meanwhile, great biocompatibility through renal metabolism also raised the possibility of clinical application. Our findings not only break the limitation of traditional QCy dyes used only for cell and molecular recognition but also provide an effective strategy for constructing efficient photosensitizers, which we believe can offer new thought in developing PSs for achieving enhanced cancer phototherapy.

## Supporting Information Available

Detailed experimental conditions, methods, synthesis, characterization, biological assays, Figures S1–S43, and Tables S1 and S2 (PDF)
