# Supplementary material for: Highly Efficient Photosensitizers with Molecular Vibrational Torsion for Cancer Photodynamic Therapy
Source: ACS Cent Sci. 2023 Jul 17;9(8):1679–91. doi: 10.1021/acscentsci.3c00611 (PMC10451034; doi:10.1021/acscentsci.3c00611)
Supplement: Supplementary file 1 — oc3c00611_si_001.pdf [file oc3c00611_si_001.pdf]

Supporting Information

Highly Efficient Photosensitizers with Molecular Vibrational Torsion for Cancer Photodynamic Therapy

Xiao Zhou[a], Chao Shi[c], Saran Long[a], Qichao Yao[a], He Ma[a], Kele Chen[a], Jianjun Du[a], Wen Sun[a], Jiangli Fan[a], Bin Liu[b], Lei Wang[b], Xiaoqiang Chen[b], Laizhi Sui[d], Yutong Zhang[d], Xiaojun Peng[ab]*

[a]State Key Laboratory of Fine Chemicals, Dalian University of Technology, 2 Linggong Road, Dalian 116024, P. R. China

[b]State Key Laboratory of Fine Chemicals, College of Materials Science and Engineering, Shenzhen University, Shenzhen 518060, P. R. China

[c]College of Chemistry and Chemical Engineering, Yantai University, Yantai 264005, P. R. China

[d]State Key Laboratory of Molecular Reaction Dynamics, Dalian Institute of Chemical Physics, Chinese Academy of Sciences, Dalian 116023, P. R. China

Email: pengxj@dlut.edu.cn

Experimental Procedures

Materials and Instrumentation

All the general chemicals used in this paper are purchased from Energy Chemical Co. and Innochem Chemical Co. unless otherwise specified. All solvents were analytically pure. Cell assay related kit including Calcein-AM/PI cell viability detection kit, DCFH-DA (2,7-dichlorofluorescein diacetate), JC-1 mitochondrial membrane potential detection kit and LDH (lactate dehydrogenase) detection kit were purchased from Beyotime Biotechnology Co. (China). All the other solvents and reagents used in this study were of analytical grade. All the BALB/c mice were purchased from Liaoning Changsheng biotechnology co., Ltd. The animal experiments have been approved by the Local Scientific Research Ethics Review Committee of the Animal Ethics Committee of Dalian University of Technology (ID no. // Ethics Approval No. 2021-043).

High resolution mass spectrometric (ESI-HRMS) were acquired on the Agilent Technologies HP1100LC/MSD MS and a Thermo Fisher LC/Q-TOF-MS instruments. ^{1}H NMR and ^{13}C NMR were obtained with Bruker Avance II 400 and Bruker Avance III 500 spectrometers. Absorption and emission spectra for all the compounds were achieved on the Agilent Technologies CARY 60 UV-Vis spectrophotometer and VAEIAN CARY Eclipse fluorescence spectrophotometer (Serial No. FL0812-M018),

respectively. Unless otherwise specified, all spectroscopic tests were performed in a quartz cell (10×10 mm). Fluorescence quantum yield of all the compounds were measured on the HAMAMATSU absolute fluorescence quantum yield spectrometer (Serial No. C11347). Triplet lifetime of compounds were acquired on LP980 laser flash photolysis spectrometer (Edinburgh Instruments, U.K.). The cell confocal laser scanning microscope (CLSM) images were all obtained by using Olympus FV3000 confocal laser scanning microscope. And the mice fluorescence imaging experiments were performed on NightOWL II LB983 small animal in vivo imaging system (German).

Synthesis of QTCy7-R

The synthesis route and method and structural characterization of QTCy7-R were shown in Scheme S1 and Figure S24-Figure S43.

Computational methods

Density functional theory (DFT) and time-dependent DFT (TD-DFT) were employed to rationalize the excited state properties of QTCy7-R. The ground state and excited state geometric configurations of the compounds were optimized by using B3LYP functional in combination with the 6-31G(d, p) basis set[1-2]. The solvent (dichloromethane) effect was included in all calculations based on the polarizable continuum model (PCM). Frequency analysis was performed to confirm that we have obtained stable structures on the potential energy surfaces. All calculations were performed on Guassian 16A unless otherwise noted. The S_1 coordinate were obtained by optimizing molecule at S_1, and S_{1V} coordinate were obtained by optimizing molecule at S_1 state with T_4 initial coordinate (The intramolecular dihedral angles were given in Figure 4, Figure S6 and Figure S7). The spin-orbital coupling (SOC) values between the S_1/S_{1V} and T_2 were calculated with ORCA 4.1[3]. Hole-Electron analysis was carried out using Multiwfn 3.8[4].

ISC rate constants calculation

The ISC rate constants from singlet to triplet states were calculated according to the Marcus theory:

$$k_{ISC} = \frac{2\pi}{\hbar}\frac{V_{SOC}^2}{\sqrt{4\pi\lambda k_B T}} e^{\left[-\frac{(-\Delta E_{ST}+\lambda)^2}{4\lambda k_B T}\right]}$$

Where k_{ISC}, $\hbar$, V_{SOC}, λ, k_B, T and ΔE_{ST} represent the ISC rate constants, reduced Planck constant, spin-orbit coupling (SOC) constant ($V_{SOC} = \langle S_1|\hat{\mathcal{H}}_{SO}|T_n\rangle$), reorganization energy, Boltzmann constant, temperature and energy gap from singlet to

triplet states ($\Delta E_{ST} = S_1 - T_n$), respectively. Boltzmann distribution function for the population of the initial vibronic manifold was considered to calculate the contribution of all initial electronic states (E_i) to the total ISC rate, each transition was weighted using the Boltzmann thermal factor:

$$P(E_i) = \frac{1}{Z} e^{[-E_i/k_B T]}$$

$$Z = \sum_i e^{[-E_i/k_B T]}$$

Where $P(E_i)$ and E_i represent Boltzmann thermal factor and the energy of each initial electronic states.

Singlet oxygen detection

Photoexcited singlet oxygen production was detected by employed 1,3-diphenylisobenzofuran (DPBF) as the indicator. The absorbance of DPBF at 415 nm was regulated to about 1.0 in DCM. And the samples were irradiated with 660 nm light for various times, then the absorption spectra of different time nodes were recorded. The singlet oxygen quantum yield (Φ_Δ) was calculated by the following equation:

$$\Phi_{sam} = \Phi_{std} \left(\frac{m_{sam}}{m_{std}}\right) \left(\frac{F_{std}}{F_{sam}}\right)$$

Where "Φ" represent the singlet oxygen quantum yield, "sam" and "std" represent QTCy7-R and MB, respectively. "m" is the slope of the absorbance of DPBF at 415 nm with time, $F=1-10^{-O.D.}$ (O.D. is the absorbance of samples at 660 nm).

Triplet lifetime measurements

The triplet life of QTCy7-R was measured by LP980 laser flash photolysis spectrometer (Edinburgh Instruments Ltd.) in combination with a Nd:YAG laser (Surelite I-10, Continuum Electro-Optics, Inc.). The oxygen in the sample was removed by aerating nitrogen for 30 min. The samples (5 μM) were excited by a 610 nm laser pulse (1 Hz, 100 mJ per pulse, fwhm ≈ 7 ns) at 300 K. The triplet state decay kinetics were measured at the maximum absorption wavelengths of each compound.

Fluorescence lifetime and femtosecond time-resolved transient absorption spectra measurements

The fluorescence lifetimes and femtosecond time-resolved transient absorption spectra of dyes were recorded on a freshly prepared samples using the time-correlated single photon counting (TCSPC) method (PicoQuant PicoHarp 300) at 300 K. The concentration is 10 μM, solvent is DCM.

Cell incubation

Hepatoma carcinoma cells (HepG-2 cells), human mammary carcinoma (MCF-7) and mouse breast cancer cells (4T1) were maintained in Dulbecco's modified Eagle's

medium (DMEM, Invitrogen) with 10 % fetal bovine serum (Invitrogen) and 1% penicillinstreptomycin. All cells were treated at 37 ºC with the humidified atmosphere containing 5% CO2 and 95% air. Before conducting cell imaging experiments, all types of cells were maintained on 35 mm glass-bottom cell dishes for 24 h.

Confocal fluorescence imaging

After culturing the cells in 35 mm glass-bottom cell dishes for 24 h, the DMEM medium was replaced with saline, then QTCy7-R was added (3 μM). The confocal fluorescence imaging was carried out and images were recorded. (λ_{ex} = 640 nm, λ_{em} = 680-780 nm). In the suborganelle localization experiments, the cells were incubated with 3 μM QTCy7-R for 90 min and then stained with LysoTracker Green DND 26 (200 nM) and MitoTracker Green FM (200 nM), respectively. After fluorescence imaging, colocalization coefficient was analyzed and recorded. (LysoTracker Green DND 26 and MitoTracker Green FM: λ_{ex} = 488 nm, λ_{em} = 500-550 nm).

Mitochondrial membrane potential detection

After culturing the cells in 35 mm glass-bottom cell dishes for 24 h, the DMEM medium was replaced with saline, the cells were divided into four groups: 1) cells incubated with 3 μM QTCy7-Me/QTCy7-Ph for 90 min under condition; 2) cells incubated with 3 μM QTCy7-Me/QTCy7-Ph for 90 min and irradiated with 660 nm light (10 mW/cm^2, 10 min). After the experiment, each group of cells was incubated with JC-1 for 20 minutes (according to the JC-1 kit instructions). Normal cells with high mitochondrial membrane potential showed red fluorescence signal (JC-1 aggregates); yet injured cells with low mitochondrial membrane potential showed green fluorescence signal (JC-1 monomer).

Intracellular ROS detection

2,7-dichlorofluorescein diacetate (DCFH-DA) Detection Kit was chosen to validate the intracellular ROS generation. After culturing the cells in 35 mm glass-bottom cell dishes for 24 h, the cells were divided into ten groups: 1) cells incubated with PBS; 2) cells incubated with PBS and irradiated with 660 nm light (10 mW/cm^2, 10 min); 3) cells incubated with QTCy7-R; 4) cells incubated with QTCy7-R and irradiated with 660 nm light (10 mW/cm^2, 10 min). After incubated with QTCy7-R or PBS, the cells were treated with DCFH-DA for 20 min. Then the cells were exposed under NIR irradiation, after which the confocal fluorescence imaging was performed. (λ_{ex} = 488 nm, λ_{em} = 500-550 nm).

Confocal imaging of cell viability

HepG2 cells were incubated in 35 mm glass-bottom cell dishes for 24 h, the

DMEM medium was replaced with saline to prevent false positive interference. Then the cells were exposed to different following treatments: 1) cells incubated with PBS; 2) cells incubated with PBS under 660 nm irradiation (20 mW/cm^2, 10 min); 3) cells incubated with QTCy7-R; 4) cells incubated with QTCy7-R under 660 nm irradiation (20 mW/cm^2, 10 min). After being different treated, the cells were stained with Calcein-AM and Propidium Iodide (PI) according to the manufacture instruction. Then the confocal fluorescence imaging was carried out and recorded with a 10 × objective lens. (Calcein-AM: λ_{ex} = 488 nm, λ_{em} = 500-550 nm; PI: λ_{ex} = 561 nm, λ_{em} = 590-640 nm).

Cytotoxicity assays

Cytotoxicity was tested by the reduction of methyl thiazolyl tetrazolium to formazan by succinic acid dehydrogenase in the mitochondria of living cells (MTT assay). HepG2 cells, MCF7 cells and 4T1 cells were seeded to 96-well microplates (Nunc, Denmark) with a density of 1 × 10^5 cells/mL cells in 100 μL DMEM medium and incubated at 37 ºC for 24 h. When the cell density reached about 70%, the QTCy7-R with different concentrations (4, 3, 2, 1.5, 1, 0.75, 0.5, 0.25, 0 μM) in PBS were added to the wells of cells in dark condition, respectively. For light groups, the cell incubation conditions were the same as those of the dark group and the cells were further incubated for 2 h, subsequently, the cells were subjected to 660 nm irradiation (20 mW/cm^2) for different time (10 min, 5 min and 2.5 min) corresponding to different light doses (12 J/cm^2, 6 J/cm^2 and 3 J/cm^2). Then all the cells were incubated for 12 h, after which the MTT solution in DMEM (0.5 mg/ml, 100 μL) was added to each well and the cells were incubated at 37 ºC for another 4 h. The MTT solution was carefully removed and 100 μL DMSO was added to dissolve formazan, and the absorbance of each well was measured by a multifunctional microplate reader at 570 nm and 630 nm. The cell viability was obtained by the following equation:

$$cell\ viability(\%) = \frac{(OD_{570} - OD_{630})_{ps}}{(OD_{570} - OD_{630})_{control}} \times 100\%$$

Where "OD" represent the absorbance at 570 nm and 630 nm, "ps" and "control" represent experimental groups and control groups, respectively.

4T1-subcutaneous tumor model and *in vivo* imaging experiments

Female BALB/c mice, 4–5 weeks of age, were selected to establish 4T1-subcutaneous tumor model. 5 × 10^6 4T1 cells were inoculated subcutaneously to their armpits. when the volume of subcutaneous tumors reached about 100 mm^3, QTCy7-Ac (200 μM, 100 μL) was injected into 4T1 tumor-bearing BALB/c mice through tail vein. And the fluorescence signals were monitored at different post-injection time by a NightOWL II LB983 small animal in vivo imaging system (German). For the images

of major organs, the mice L) was intravenously injected with QTCy7-Ac (200 μM, 100 μL) 2 hours in advance were killed and the main organs and tumors were removed for imaging experiment.

Tumor suppression experiments of 4T1-subcutaneous tumor model

To confirm the in vivo PDT efficacy of QTCy7-Ac, all the BALB/c mice were divided into 6 groups: 1) saline injection; 2) saline injection with 660 nm irradiation (100 mW/cm^2, 15 min); 3) QTCy7-Ac (200 μM, 100 μL) intratumoral injection; 4) QTCy7-Ac (200 μM, 100 μL) intratumoral injection with 660 nm irradiation (100 mW/cm^2, 15 min); 5) QTCy7-Ac (200 μM, 100 μL) intravenous injection; 6) QTCy7-Ac (200 μM, 100 μL) intravenous injection with 660 nm irradiation (100 mW/cm^2, 15 min); When the volume of subcutaneous tumors reached about 100 mm^3, mice were injected with PBS or QTCy7-Ac, respectively. After 2 h post-injection, tumor region was irradiated with 650 nm light. In the following half month, the tumor volume of all the mice was measured every two days by a vernier caliper. The volume of tumors was calculated by the following formula:

$$V = \frac{a \times b^2}{2}$$

Where "V" represent the tumor volume, "a" represent greatest longitudinal diameter (length) and "b" represent the greatest transverse diameter (width).

***In Vivo* Biosafety Assay**

The *in vivo* biocompatibility was evaluated by using measurement mice body weight and H&E slice histological analysis. After the PDT treatment, the mice were euthanized, and main organs including heart, liver, spleen, lung, kidneys and tumors were harvested for histological analysis by means of hematoxylin-eosin (H&E) staining.

Statistical analysis

Data were expressed as mean ± standard deviation. Student's t test was used to evaluate the statistical significance. P values < 0.05 were regarded statistically significant (*p < 0.05, **p < 0.01, ***p < 0.001, ****p < 0.0001).

Synthesis routes of QTCy7-R

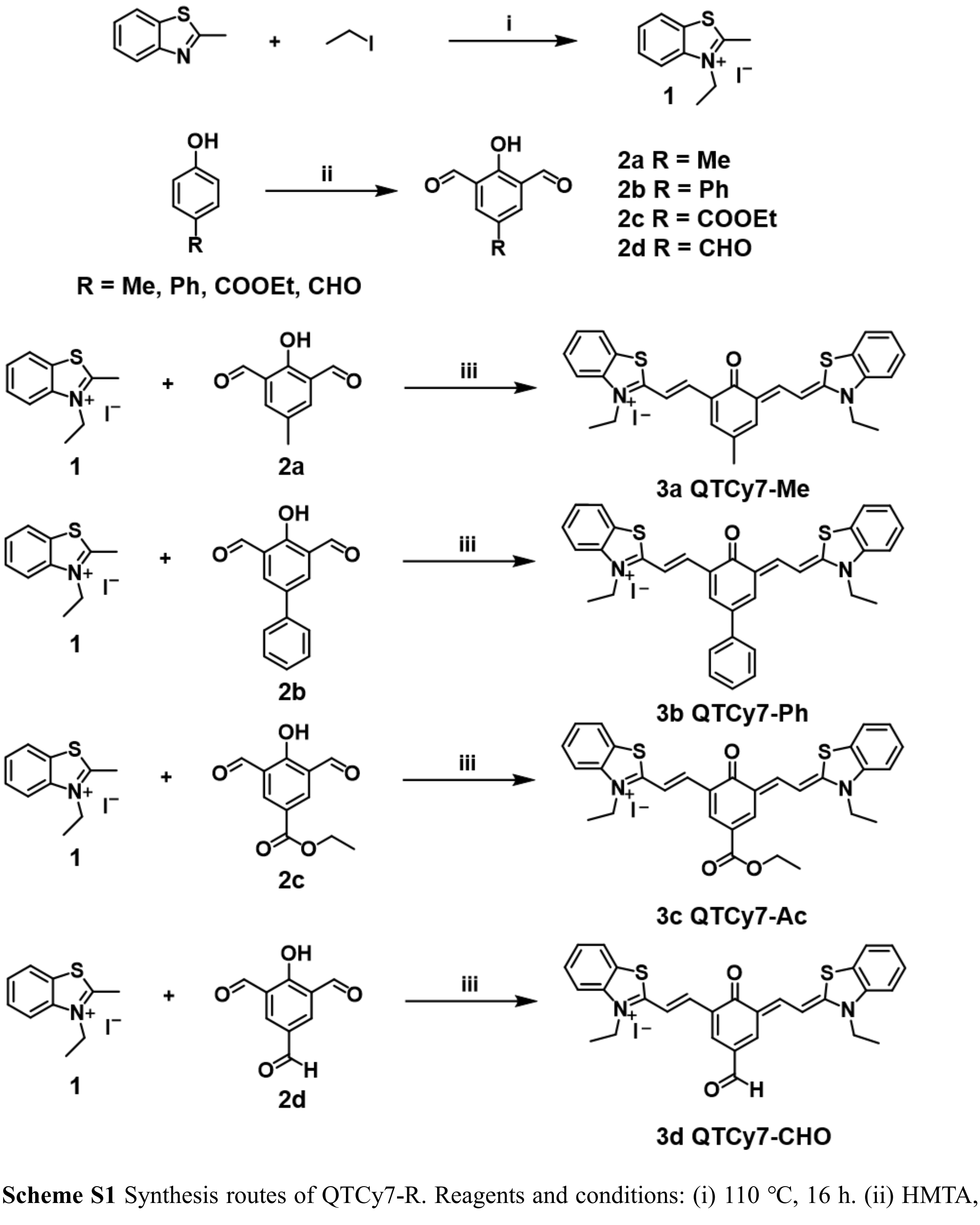

Scheme S1 Synthesis routes of QTCy7-R. Reagents and conditions: (i) 110 °C, 16 h. (ii) HMTA, TFA, reflux, 24 h. HCl, H_2O, 80 °C, 3h. (iii) MeOH, NaOAc, 70 °C, 5h.

TCy7 was prepared according to the literature methods[5].

Synthesis of compound 1

2-methylbenzothiazole (1.5 g, 10 mmol) and iodoethane (3.12 g, 20 mmol) were stirred in a quartz seal tube and heated to 110 °C for 16 hours. Subsequently, the reaction mixure was cool to room temperature. The precipitate was filtered and dried in vacuum

to obtain a compound 1 as a white solid. (Yield: 86%)

Synthesis of compound 2a

4-methylphenol (1 g, 9.25 mmol) and hexamethylenetetramine (4.2 g, 30 mmol) were dissolved in 10 mL trifluoroacetic acid. The reaction mixture was stirred at 100 °C for 24 h under N_2 atmosphere. Subsequently, the mixture was cooled down to 70 °C and 50 mL HCl (4 M) was added carefully. After 3 h reaction, the product was cooled to room temperature, the precipitate was filtered and dried in vacuum to obtain a pale yellow solid. If no precipitation formed, the product was extracted with dichloromethane and the solvent was removed under vacuum. The residue was purified by column chromatography with petroleum-ether/EtOAc (5:1) as eluent to afford a pale yellow solid. (Yield: 74%) ^1H NMR (500 MHz, DMSO-d_6) δ 11.39 (s, 1H), 10.22 (s, 2H), 7.86 (s, 2H), 2.34 (s, 3H) ppm. ESI-MS ($C_9H_7O_3$) m/z: $[M-H]^-$ calcd 163.0401, found 163.0401.

Synthesis of compound 2b

4-phenylphenol (1.36 g, 8.0 mmol) and hexamethylenetetramine (3.4 g, 24 mmol) were dissolved in 10 mL trifluoroacetic acid. The reaction mixture was stirred at 110 °C for 48 h under N_2 atmosphere. Subsequently, the mixture was cooled down to 70 °C and 50 mL HCl (4 M) was added carefully. After 3 h reaction, the product was cooled to room temperature, the precipitate was filtered and dried in vacuum to obtain a pale yellow solid. If no precipitation formed, the product was extracted with dichloromethane and the solvent was removed under vacuum. The residue was purified by column chromatography with petroleum-ether/EtOAc (6:1) as eluent to afford a pale yellow solid. (Yield: 67%) ^1H NMR (500 MHz, DMSO-d_6) δ 11.61 (s, 1H), 10.32 (s, 2H), 8.34 (s, 2H), 7.72 (d, J = 7.8 Hz, 2H), 7.51 (t, J = 7.7 Hz, 2H), 7.41 (t, J = 7.4 Hz, 1H) ppm. ESI-MS ($C_{14}H_9O_3$) m/z: $[M-H]^-$ calcd 225.0557, found 225.0555.

Synthesis of compound 2c

Ethyl 4-hydroxybenzoate (1 g, 6.02 mmol) and hexamethylenetetramine (2.8 g, 20 mmol) were dissolved in 10 mL trifluoroacetic acid. The reaction mixture was stirred at 100 °C for 48 h under N_2 atmosphere. Subsequently, the mixture was cooled down to 70 °C and 50 mL HCl (4 M) was added carefully. After 3 h reaction, the product was cooled to room temperature, the precipitate was filtered and dried in vacuum to obtain a pale yellow solid. If no precipitation formed, the product was extracted with dichloromethane and the solvent was removed under vacuum. The residue was purified by column chromatography with petroleum-ether/EtOAc (3:1) as eluent to afford a pale yellow solid. (Yield: 60%) ^1H NMR (500 MHz, DMSO-d_6) δ 10.29 (s, 2H), 8.53 (s,

2H), 4.35 (q, J = 7.1 Hz, 2H), 1.35 (t, J = 7.1 Hz, 3H) ppm. ESI-MS ($C_{11}H_9O_5$) m/z: $[M - H]^-$ calcd 221.0455, found 221.0459.

Synthesis of compound 2d

4-Hydroxybenzaldehyde (1 g, 10.63 mmol) and hexamethylenetetramine (4.2 g, 30 mmol) were dissolved in 10 mL trifluoroacetic acid. The reaction mixture was stirred at 110 °C for 24 h then 150 °C for 3 h under N_2 atmosphere. Subsequently, the mixture was cooled down to 70 °C and 50 mL HCl (4 M) was added carefully. After 3 h reaction, the product was cooled to room temperature, the precipitate was filtered and dried in vacuum to obtain a pale yellow solid. If no precipitation formed, the product was extracted with dichloromethane and the solvent was removed under vacuum. The residue was purified by column chromatography with petroleum-ether/EtOAc (2:1) as eluent to afford a pale yellow solid. (Yield: 34%) ^{1}H NMR (500 MHz, DMSO-d_6) δ 10.32 (s, 2H), 10.00 (s, 1H), 8.53 (s, 2H) ppm. ESI-MS ($C_9H_5O_4$) m/z: $[M - H]^-$ calcd 177.0193, found 177.0199.

Synthesis of compound 3a (QTCy7-Me)

Compound 1 (152 mg, 0.5 mmol) and compound 2a (33 mg, 0.2 mmol) were dissolved in 15 mL CH_3OH, then sodium acetate (50 mg, 0.61 mmol) was added and the mixture was stirred at 70 °C for 5 h under N_2 atmosphere. Subsequently, the crude product was evaporated in vacuum and the residue was purified by column chromatography with CH_2Cl_2/CH_3OH (15:1) as eluent to obtain a blue solid with metallic luster. (Yield: 83%) ^{1}H NMR (500 MHz, DMSO-d_6) δ 8.34 (d, J = 8.0 Hz, 2H), 8.20 (t, J = 11.7 Hz, 4H), 8.10 (d, J = 15.1 Hz, 2H), 7.84 (s, 2H), 7.80 (t, J = 8.1 Hz, 2H), 7.70 (t, J = 7.7 Hz, 2H), 4.82 (dd, J = 14.0, 6.8 Hz, 4H), 2.29 (s, 3H), 1.49 (t, J = 7.2 Hz, 6H) ppm. ^{13}C NMR (101 MHz, DMSO-d_6) δ 172.48, 171.84, 148.12, 141.38, 139.59, 139.51, 129.38, 127.64, 127.57, 125.84, 124.41, 115.94, 107.16, 43.92, 20.39, 14.04 ppm. ESI-MS ($C_{29}H_{27}IN_2OS_2$) m/z: $[M - I]^+$ calcd 483.1559, found 483.1554.

Synthesis of compound 3b (QTCy7-Ph)

Compound 1 (152 mg, 0.5 mmol) and compound 2b (45 mg, 0.2 mmol) were dissolved in 15 mL CH_3OH, then sodium acetate (50 mg, 0.61 mmol) was added and the mixture was stirred at 70 °C for 5 h under N_2 atmosphere. Subsequently, the crude product was evaporated in vacuum and the residue was purified by column chromatography with CH_2Cl_2/CH_3OH (15:1) as eluent to obtain a blue solid with metallic luster. (Yield: 89%) ^{1}H NMR (400 MHz, DMSO-d_6) δ 8.41 (d, J = 14.6 Hz, 2H), 8.35 – 8.24 (m, 4H), 8.22 – 8.06 (m, 4H), 7.76 (t, J = 7.3 Hz, 4H), 7.66 (t, J = 7.4 Hz, 2H), 7.47 (t, J = 6.9 Hz, 2H), 7.31 (t, J = 7.3 Hz, 1H), 4.90 – 4.58 (m, 4H), 1.49 (t, J = 6.2 Hz, 6H) ppm. ^{13}C

NMR (101 MHz, DMSO-d_6) δ 177.29, 172.30, 149.43, 141.41, 139.89, 137.47, 129.39, 129.19, 127.66, 127.59, 126.57, 126.44, 125.90, 124.42, 123.88, 115.97, 107.35, 43.95, 13.99 ppm. ESI-MS ($C_{34}H_{29}IN_2OS_2$) m/z: $[M - I]^+$ calcd 545.1716, found 545.1708.

Synthesis of compound 3c (QTCy7-Ac)

Compound 1 (152 mg, 0.5 mmol) and compound 2c (45 mg, 0.2 mmol) were dissolved in 15 mL CH_3OH, then sodium acetate (50 mg, 0.61 mmol) was added and the mixture was stirred at 70 °C for 5 h under N_2 atmosphere. Subsequently, the crude product was evaporated in vacuum and the residue was purified by column chromatography with CH_2Cl_2/CH_3OH (15:1) as eluent to obtain a blue solid with metallic luster. (Yield: 71%) ^{1}H NMR (400 MHz, DMSO-d_6) δ 8.54 (d, J = 14.8 Hz, 2H), 8.40 – 8.15 (m, 8H), 7.80 (t, J = 7.5 Hz, 2H), 7.70 (t, J = 7.5 Hz, 2H), 4.78 (q, J = 6.7 Hz, 4H), 4.31 (q, J = 6.6 Hz, 2H), 1.50 (t, J = 6.4 Hz, 6H), 1.36 (t, J = 6.4 Hz, 3H) ppm. ^{13}C NMR (101 MHz, DMSO-d_6) δ 178.67, 173.03, 165.96, 149.58, 141.37, 140.04, 129.52, 127.98, 127.91, 125.41, 124.52, 116.29, 112.46, 109.01, 60.39, 44.28, 15.02, 14.05 ppm. ESI-MS ($C_{31}H_{29}IN_2OS_2$) m/z: $[M - I]^+$ calcd 541.1614, found 541.1617.

Synthesis of compound 3d (QTCy7-CHO)

Compound 1 (152 mg, 0.5 mmol) and compound 2d (36 mg, 0.2 mmol) were dissolved in 15 mL CH_3OH, then sodium acetate (50 mg, 0.61 mmol) was added and the mixture was stirred at 70 °C for 5 h under N_2 atmosphere. Subsequently, the crude product was evaporated in vacuum and the residue was purified by column chromatography with CH_2Cl_2/CH_3OH (15:1) as eluent to obtain a blue solid with metallic luster. (Yield: 71%) ^{1}H NMR (400 MHz, DMSO-d_6) δ 9.66 (s, 1H), 8.43 (d, J = 15.1 Hz, 2H), 8.36 – 8.16 (m, 8H), 7.80 (t, J = 7.4 Hz, 2H), 7.70 (t, J = 7.3 Hz, 2H), 4.80 (d, J = 6.5 Hz, 4H), 1.50 (t, J = 6.3 Hz, 6H) ppm. ^{13}C NMR (101 MHz, DMSO-d_6) δ 189.02, 179.03, 172.88, 148.85, 141.40, 139.75, 129.59, 128.10, 127.96, 125.81, 124.60, 121.21, 116.39, 109.31, 44.35, 14.17 ppm. ESI-MS ($C_{29}H_{25}IN_2OS_2$) m/z: $[M - I]^+$ calcd 497.1352, found 497.1345.

Supplementary data

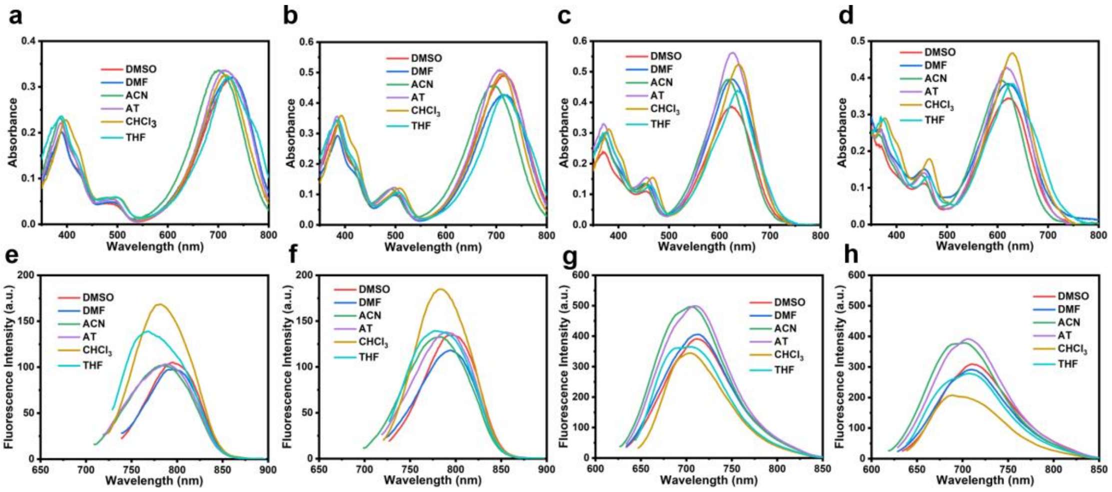

Figure S1. The UV−vis absorption spectra and fluorescence emission spectra of (a) (e) QTCy7-Me, (b) (f) QTCy7-Ph, (c) (g) QTCy7-Ac and (d) (h) QTCy7-CHO in different solvents, the concentration was 10 μM.

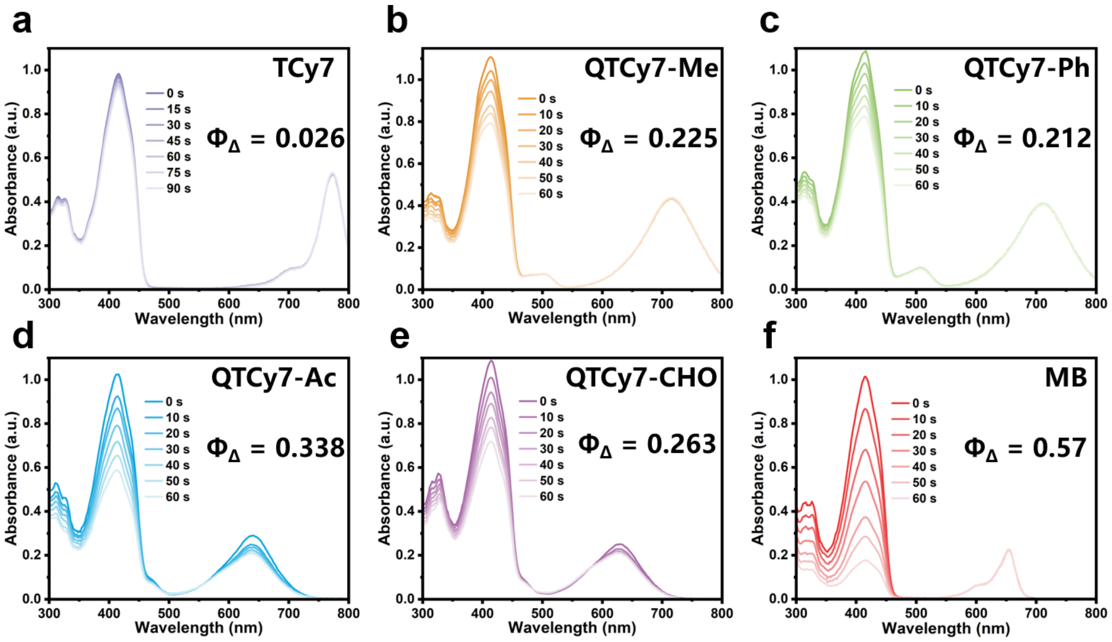

Figure S2. The DPBF degradation induced by (a) TCy7, (b) QTCy7-Me, (c) QTCy7-Ph, (d) QTCy7-Ac, (e) QTCy7-CHO and (f) MB under 660 nm light irradiation (2 mW/cm^2) of different durations.

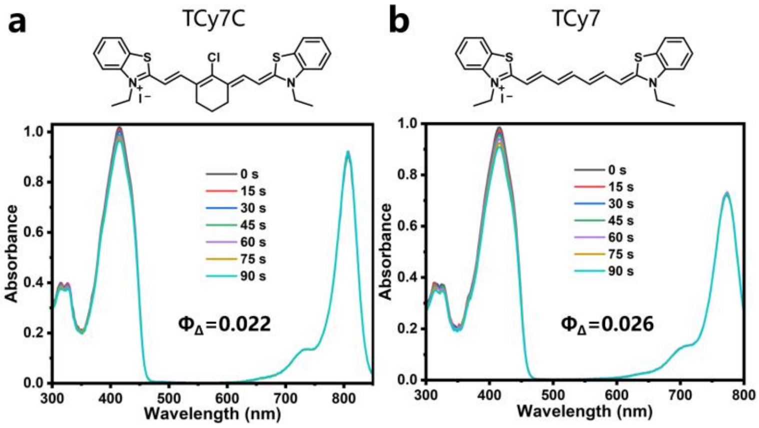

Figure S3. The DPBF degradation induced by rigidified TCy7C and free TCy7 under 660 nm light irradiation (2 mW/cm^2) of different durations.

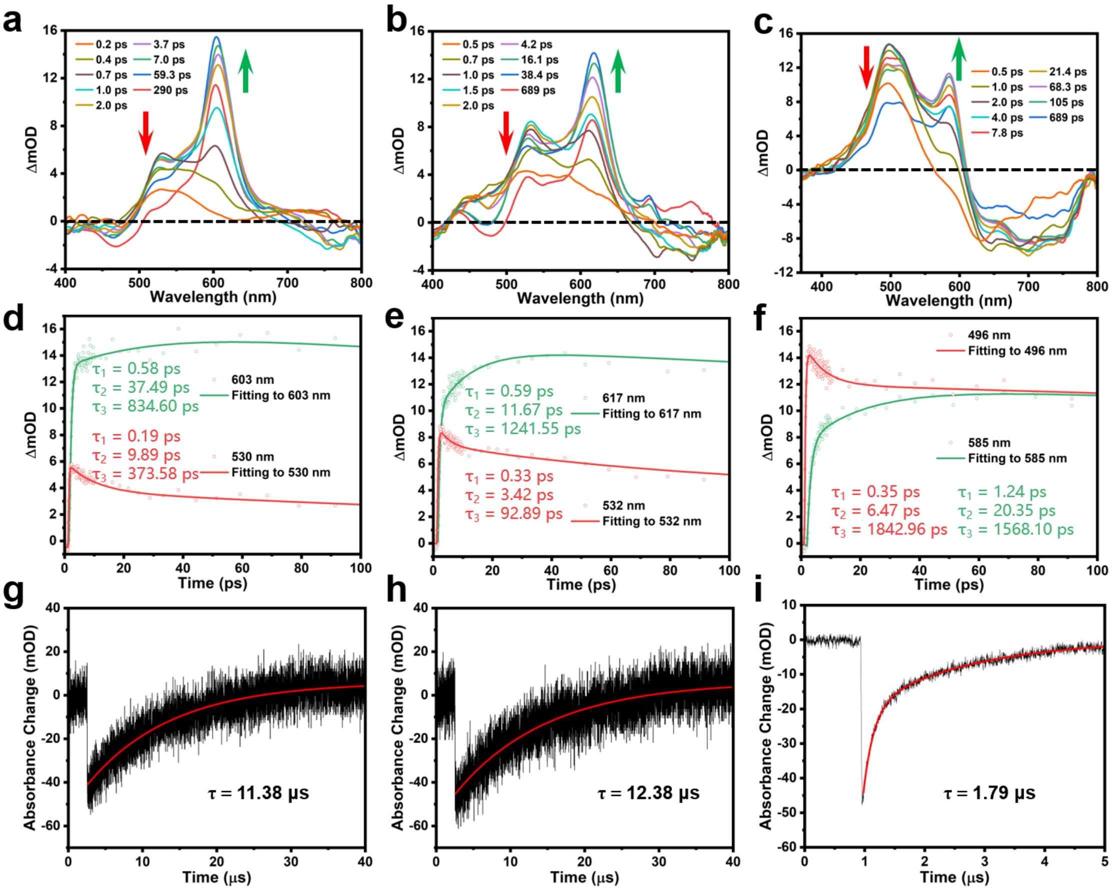

Figure S4. Femtosecond transient absorption spectroscopy (fs-TA) analysis for (a) QTCy7-Me, (b) QTCy7-Ph and (c) QTCy7-CHO at different pump probe delay times, different color lines represent spectra at different times, (λ_{ex} = 350 nm). Kinetic traces and fitting lines of (d) QTCy7-Me, (e) QTCy7-Ph and (f) QTCy7-CHO taken through the representative ESA wavelength. Kinetic traces of triplet state of (g) QTCy7-Me, (h) QTCy7-Ph and (i) QTCy7-CHO in deaerated dichloromethane at 710 nm, 714 nm and 610 nm, respectively. λ_{ex} = 610 nm.

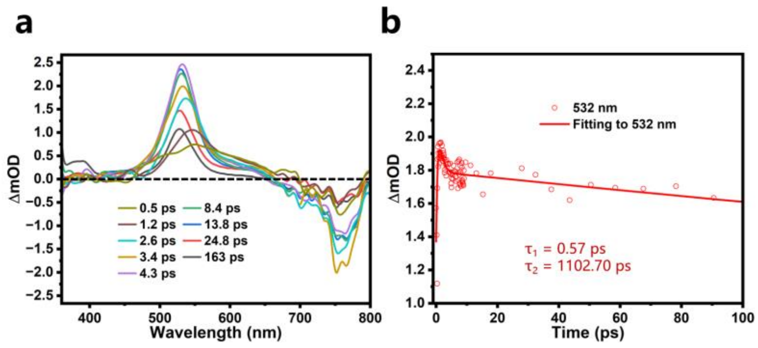

Figure S5. Femtosecond transient absorption spectroscopy (fs-TA) analysis and kinetic trace and fitting line for TCy7 (λ_{ex} = 350 nm).

	E(QTCy7-Me)/eV	E(QTCy7-Ph)/eV	E(QTCy7-Ac)/eV	E(QTCy7-CHO)/eV
S_1 coordinate	1.6822	1.6325	1.8250	1.8445
S_{1V} coordinate	1.6802	1.6343	1.8334	1.8444
$\Delta E(S_{1V}-S_1)$	0.0020	0.0018	0.0084	-0.0001

Table S1. The energy of S_1 of for QTCy7-R at S_1 and S_{1V} coordinates and their energy difference.

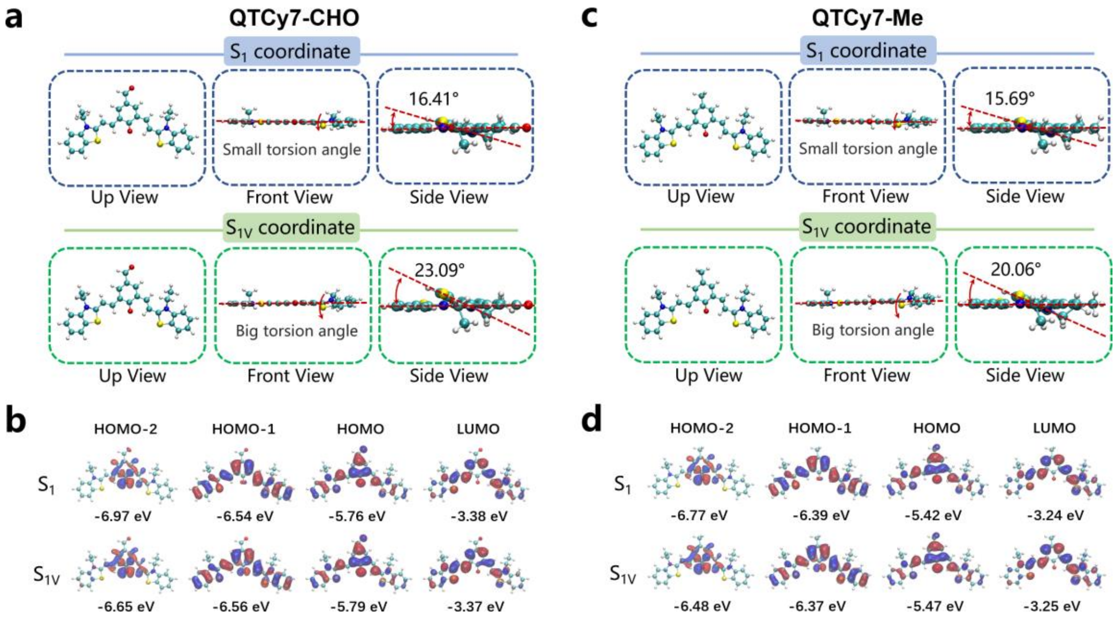

Figure S6. (a) The views from different locations (up, front and side) of QTCy7-CHO and their torsion angles in S_1 and S_{1V} coordinates. (b) The frontier molecular orbital (FMO) and the corresponding energy of QTCy7-CHO in S_1 and S_{1V} coordinates. (c) The views from different locations (up, front and side) of QTCy7-Me and their torsion angles in S_1 and S_{1V} coordinates. (d) The frontier molecular orbital (FMO) and the corresponding energy of QTCy7-Me in S_1 and S_{1V} coordinates.

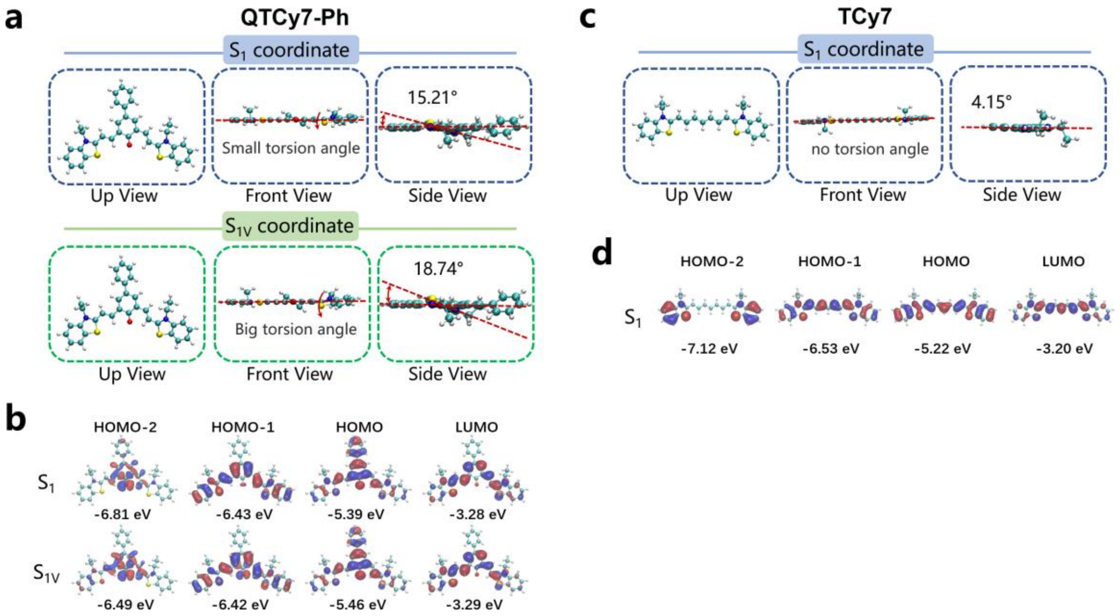

Figure S7. (a) The views from different locations (up, front and side) of QTCy7-Ph and their torsion angles in S_1 and S_{1V} coordinates. (b) The frontier molecular orbital (FMO) and the corresponding energy of QTCy7-Ph in S_1 and S_{1V} coordinates. (c) The views from different locations (up, front and side) of TCy7 and their torsion angles in S_1 and S_{1V} coordinates. (d) The frontier molecular orbital (FMO) and the corresponding energy of TCy7 in S_1 and S_{1V} coordinates.

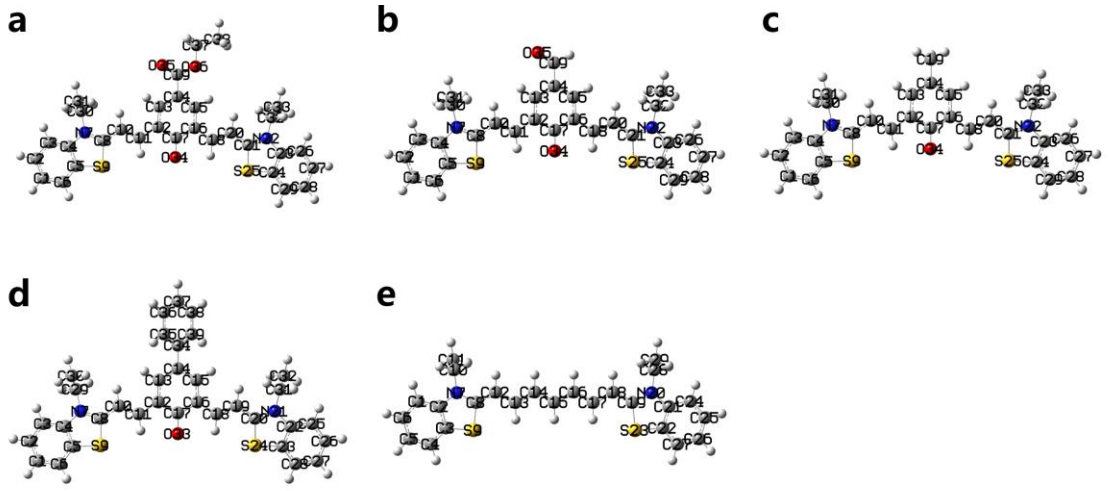

Figure S8. The atomic serial number of (a) QTCy7-Ac, (b) QTCy7-CHO, (c) QTCy7-Me, (d) QTCy7-Ph and (e) TCy7 for the corresponding heat maps.

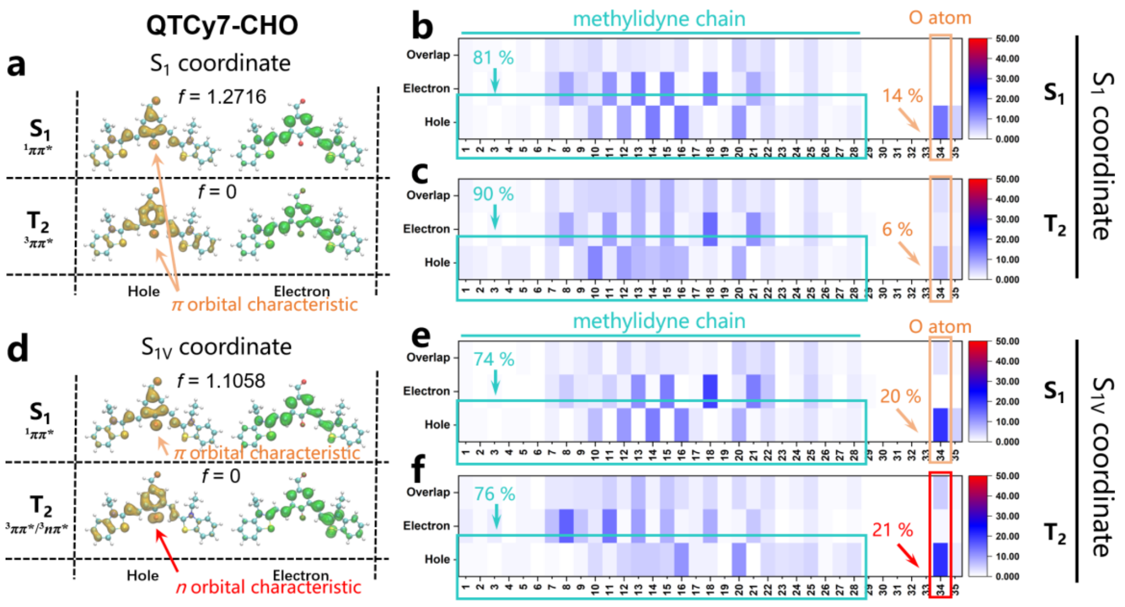

Figure S9. (a) The hole-electron distribution of QTCy7-CHO at S_1 and T_2 in S_1 coordinate. (b) (c) The heat map of hole-electron distribution of QTCy7-CHO at S_1 and T_2 in S_1 coordinate. (d) The hole-electron distribution of QTCy7-CHO at S_1 and T_2 in S_{1V} coordinate. (e) (f) The heat map of hole-electron distribution of QTCy7-CHO at S_1 and T_2 in S_{1V} coordinate.

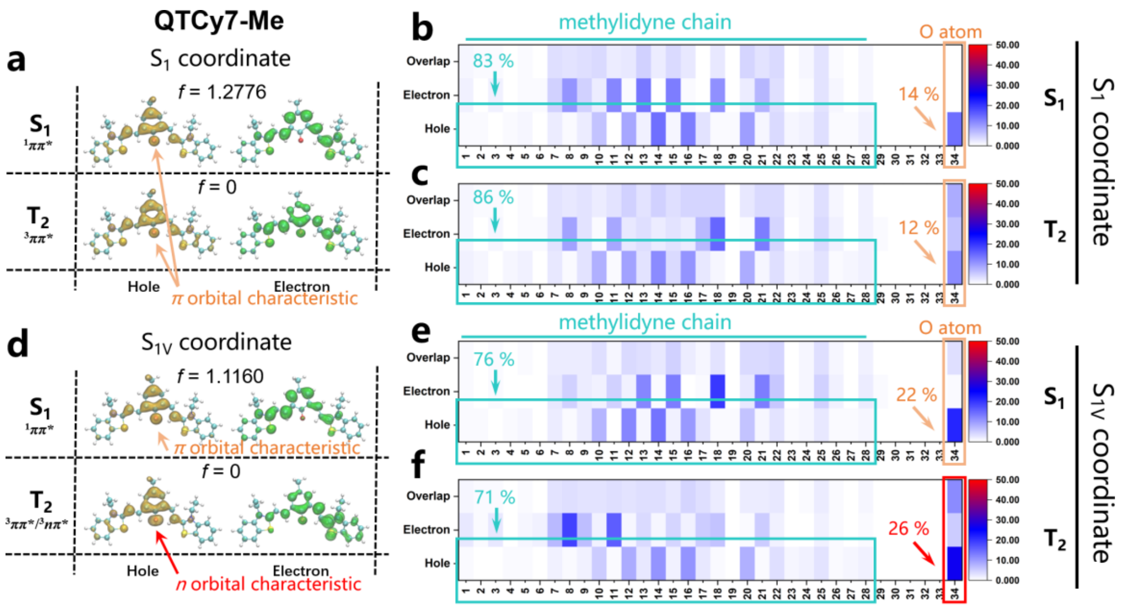

Figure S10. (a) The hole-electron distribution of QTCy7-Me at S_1 and T_2 in S_1 coordinate. (b) (c) The heat map of hole-electron distribution of QTCy7-Me at S_1 and T_2 in S_1 coordinate. (d) The hole-electron distribution of QTCy7-Me at S_1 and T_2 in S_{1V} coordinate. (e) (f) The heat map of hole-electron distribution of QTCy7-Me at S_1 and T_2 in S_{1V} coordinate.

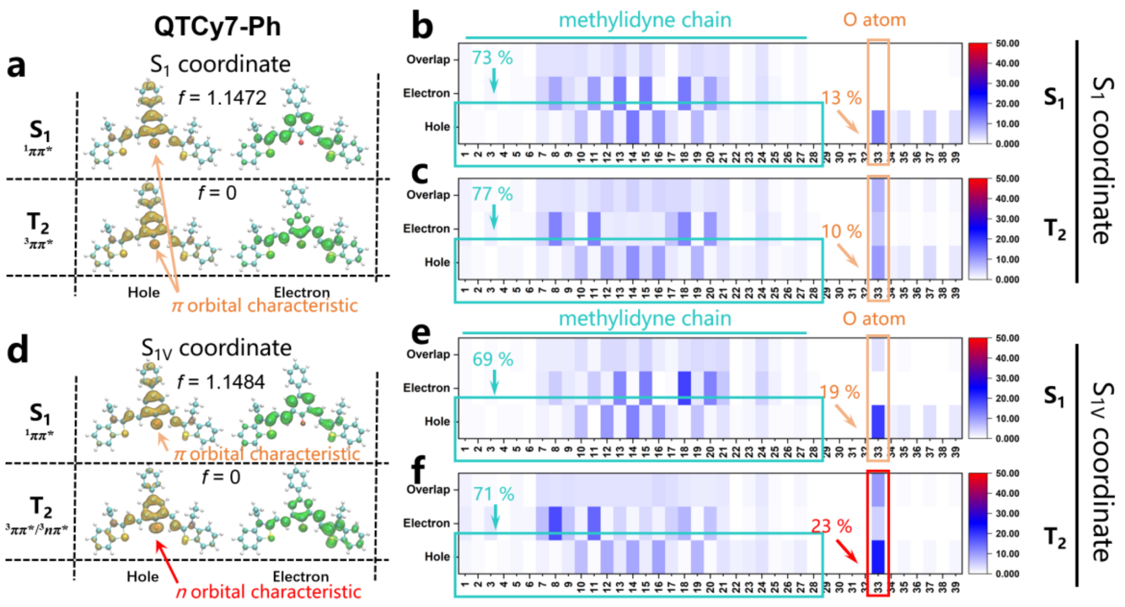

Figure S11. (a) The hole-electron distribution of QTCy7-Ph at S_1 and T_2 in S_1 coordinate. (b) (c) The heat map of hole-electron distribution of QTCy7-Ph at S_1 and T_2 in S_1 coordinate. (d) The hole-electron distribution of QTCy7-Ph at S_1 and T_2 in S_{1V} coordinate. (e) (f) The heat map of hole-electron distribution of QTCy7-Ph at S_1 and T_2 in S_{1V} coordinate.

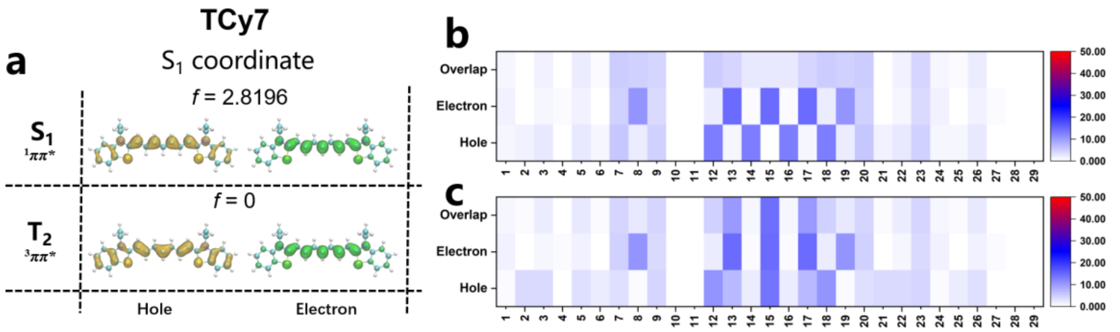

Figure S12. (a) The hole-electron distribution of TCy7 at S_1 and T_2 in S_1 coordinate. (b) (c) The heat map of hole-electron distribution of TCy7 at S_1 and T_2 in S_1 coordinate.

compounds	ISC	ΔE_{ST}(eV)	λ(eV)	SOC(cm^{-1})	k_{ISC}(s^{-1})	Total k_{ISC}(s^{-1})
QTCy7-Me	S_1-T_2	0.04	0.06	0.23	5.21×10^7	1.20×10^8
	S_{1V}-T_2	0.06	0.02	0.47	1.86×10^8	
QTCy7-Ph	S_1-T_2	-0.02	0.05	0.21	1.97×10^7	1.03×10^8
	S_{1V}-T_2	-0.02	0.03	0.52	1.86×10^8	
QTCy7-Ac	S_1-T_2	0.18	0.16	0.36	8.14×10^7	1.76×10^8
	S_{1V}-T_2	0.19	0.21	0.70	2.70×10^8	
QTCy7-CHO	S_1-T_2	0.20	0.17	0.42	1.05×10^8	1.26×10^8
	S_{1V}-T_2	0.22	0.19	0.51	1.47×10^8	
TCy7	S_1-T_2	-0.15	0.32	0.07	2.84×10^4	2.84×10^4

Table S2. The energy gap (ΔE_{ST}), reorganization energy (λ), spin-orbit coupling (SOC) and ISC rate

constants (k_{ISC}).

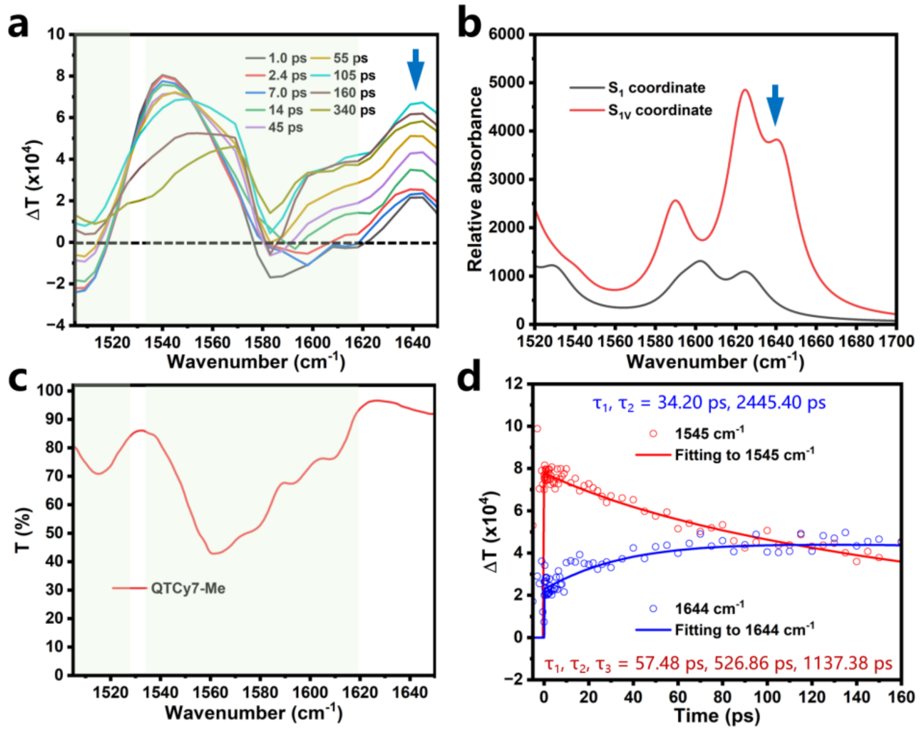

Figure S13. (a) TRIR spectra for QTCy7-Me at different pump probe delay times, different color lines represent spectra at different times. (b) Simulated IR spectra for QTCy7-Me at S_1 and S_{1V} coordinates. (c) FTIR spectrum for QTCy7-Me. (d) Kinetic traces and fitting lines of QTCy7-Me taken through 1545 cm^{-1} and 1644 cm^{-1}.

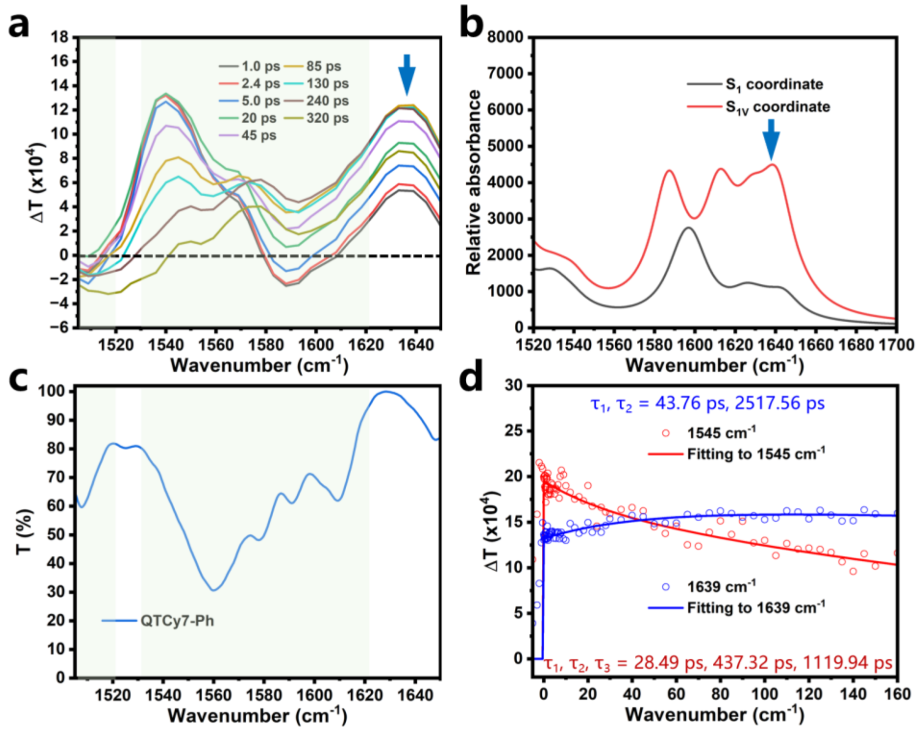

Figure S14. (a) TRIR spectra for QTCy7-Ph at different pump probe delay times, different color lines represent spectra at different times. (b) Simulated IR spectra for QTCy7-Ph at S_1 and S_{1V} coordinates. (c) FTIR spectrum for QTCy7-Ph. (d) Kinetic traces and fitting lines of QTCy7-Ph taken through 1545 cm^{-1} and 1639 cm^{-1}.

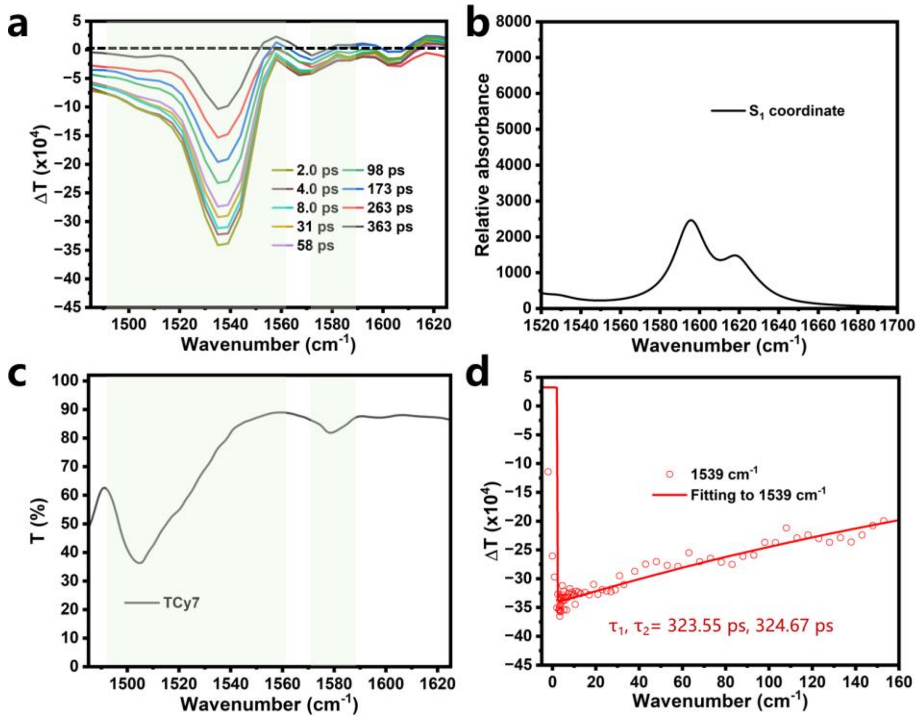

Figure S15. (a) TRIR spectra for TCy7 at different pump probe delay times, different color lines represent spectra at different times. (b) Simulated IR spectra for TCy7 at S_1 coordinate. (c) FTIR spectrum for TCy7. (d) Kinetic traces and fitting lines of TCy7 taken through 1539 cm^{-1}.

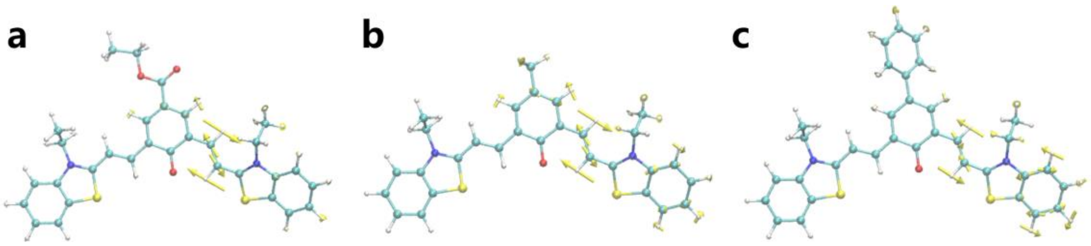

Figure S16. The molecular structures and vibrational vectors (yellow arrows) of (a) 1644 cm^{-1} of QTCy7-Ac, (b) 1643 cm^{-1} of QTCy7-Me and (c) 1641 cm^{-1} of QTCy7-Ph at their S_{1V} coordinates.

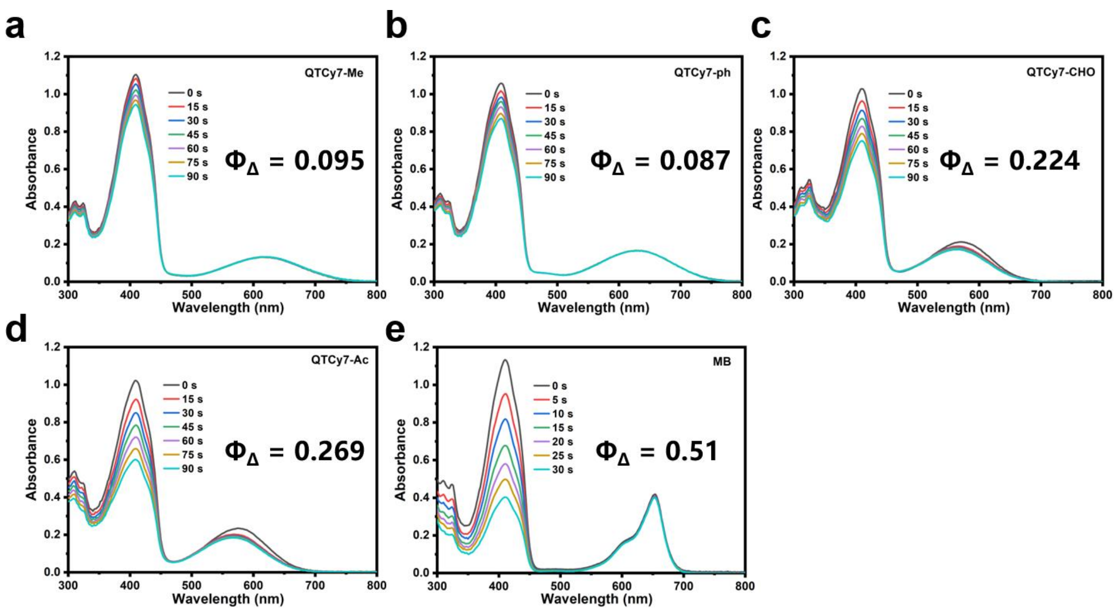

Figure S17. The DPBF degradation induced by (a) QTCy7-Me, (b) QTCy7-Ph, (c) QTCy7-CHO, (d) QTCy7-Ac and (e) MB under 630 nm light irradiation (2 mW/cm^2) of different durations in MeOH.

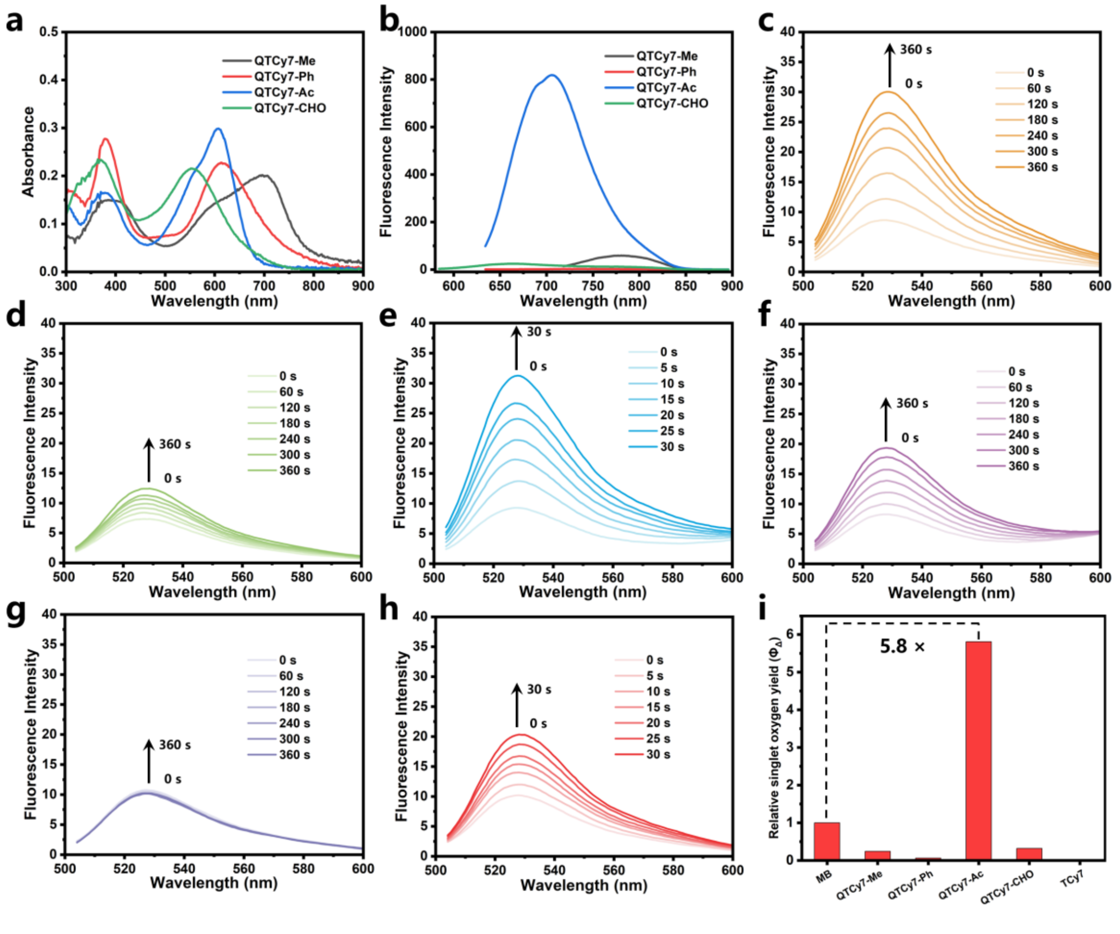

Figure S18. (a) The UV-vis absorption spectra and (b) fluorescence emission spectra of QTCy7-R (10 μM) in PBS. (c-h) The SOSG fluorescence enhancement induced by (c) QTCy7-Me, (d)

QTCy7-Ph, (e) QTCy7-Ac, (f) QTCy7-CHO, (g) TCy7 and (h) MB under 660 nm irradiation (4 mW/cm^2) in PBS. (i) The relative singlet oxygen yield of different compounds in PBS.

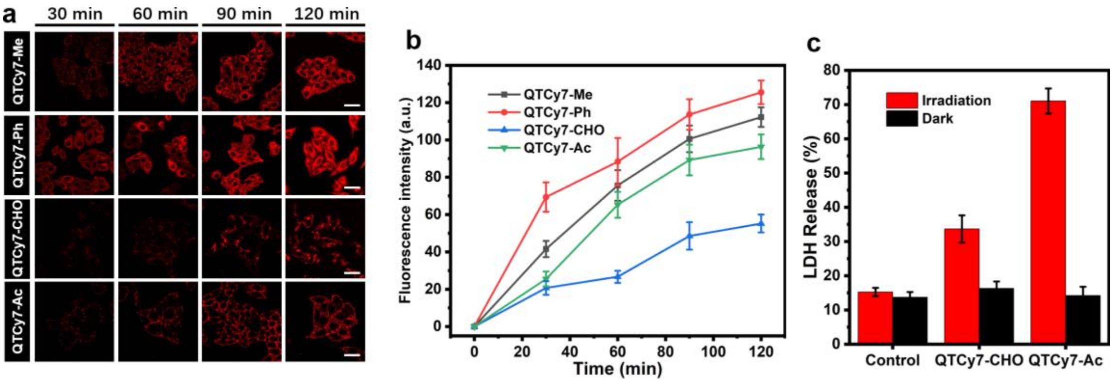

Figure S19. (a) The cellular uptake of QTCy7-R in HepG2 cells (QTCy7-Me/QTCy7-Ph: λ_{ex} = 640 nm, λ_{em} = 700-800 nm, QTCy7-CHO/QTCy7-Ac: λ_{ex} = 640 nm, λ_{em} = 650-750 nm,scale bars: 20 μm). (b) The fluorescence intensity of QTCy7-R in cell at different time. (c) The LDH release rate of cells under different treatment conditions.

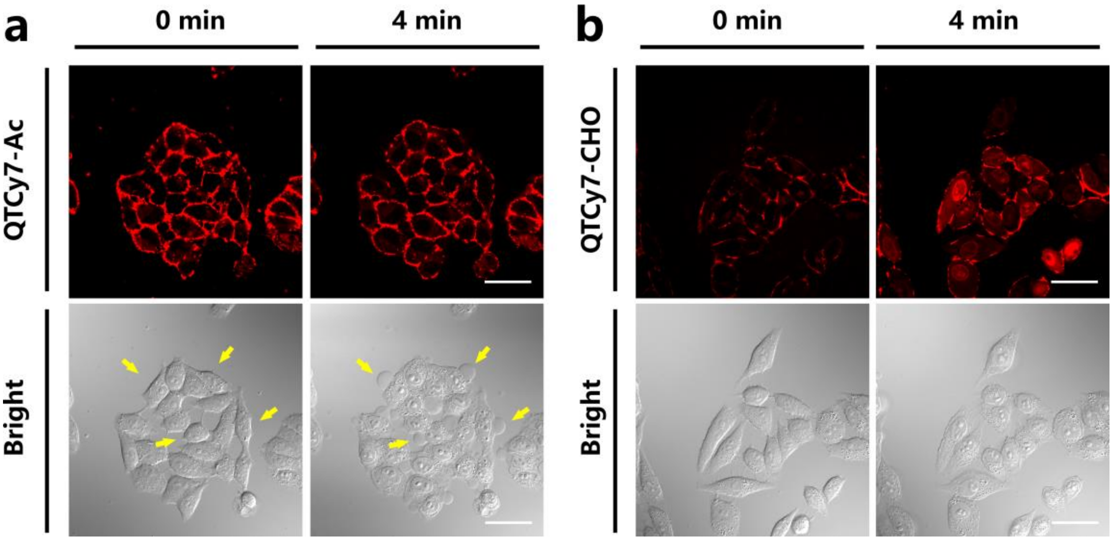

Figure S20. The enlarged images of (a) QTCy7-Ac and (b) QTCy7-CHO caused cell membrane destruction at 0 min and 4 min. Yellow arrows indicate the formation of distinct bubble-like structures on cell membrane. (λ_{ex} = 640 nm, λ_{em} = 650-750 nm, scale bars: 20 μm).

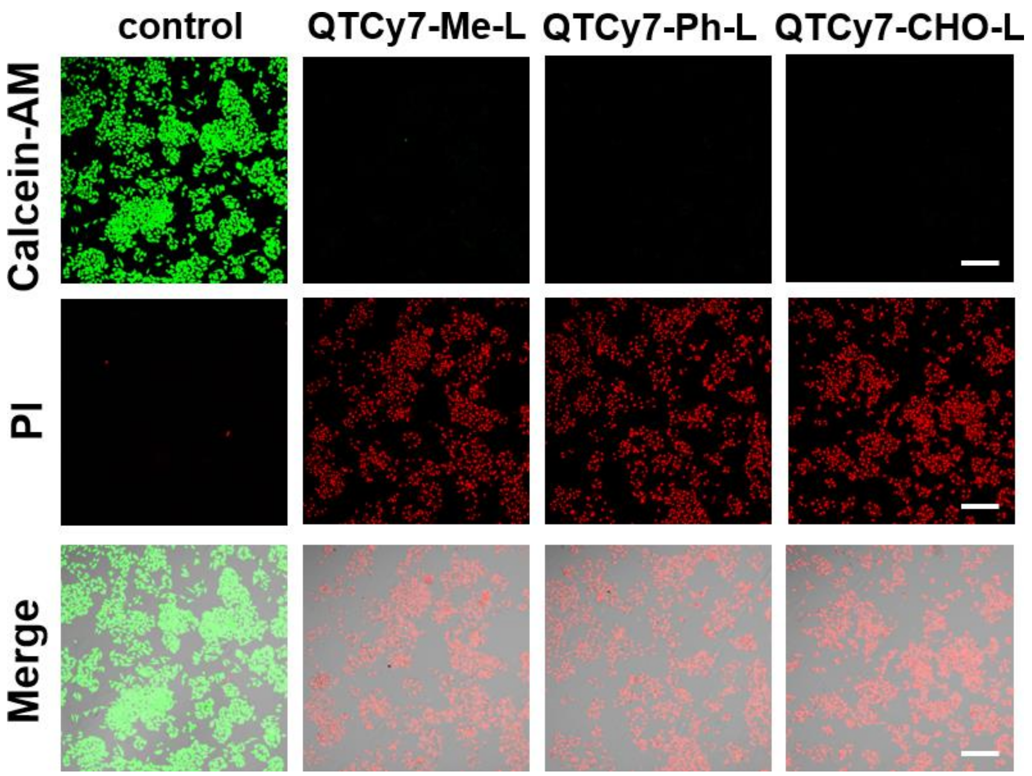

Figure S21. Cell viability detection images measured by Calcein-AM/PI under different treatment conditions. (Calcein-AM: λ_{ex} = 488 nm, λ_{em} = 500−550 nm; PI: λ_{ex} = 561 nm, λ_{em} = 580-630 nm, scale bars: 120 μm).

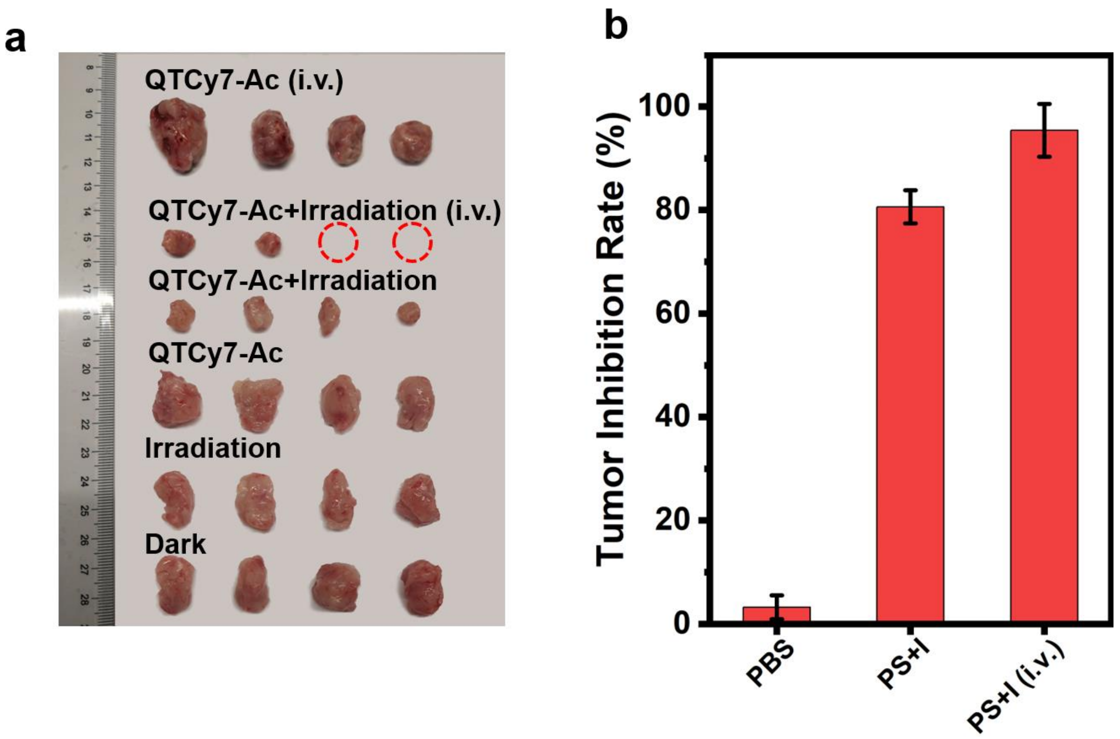

Figure S22. (a) The picture of isolated tumors at different groups. (b) The tumor inhibition rate in different groups.

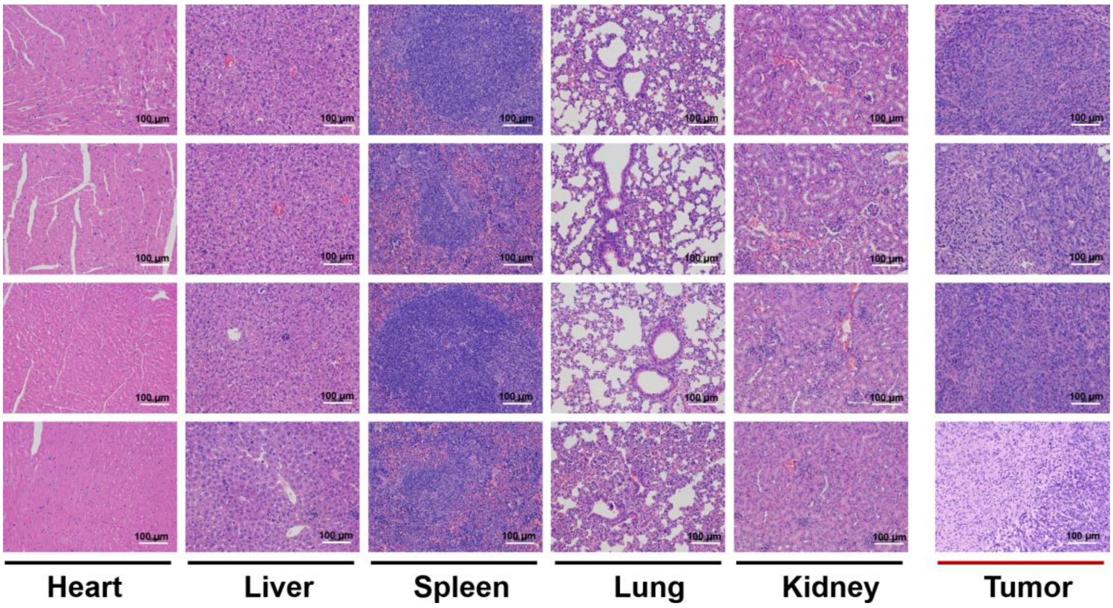

Figure S23. H&E staining assays of main organs and tumors at Dark group (first line), Irradiation group (second line), QTCy7-Ac group (third line) and QTCy7-Ac+irradiation group (final line) after 16 days of treatment. (Scale bar = 100 μm)

ESI-MS, ^{1}H NMR and ^{13}C NMR spectra

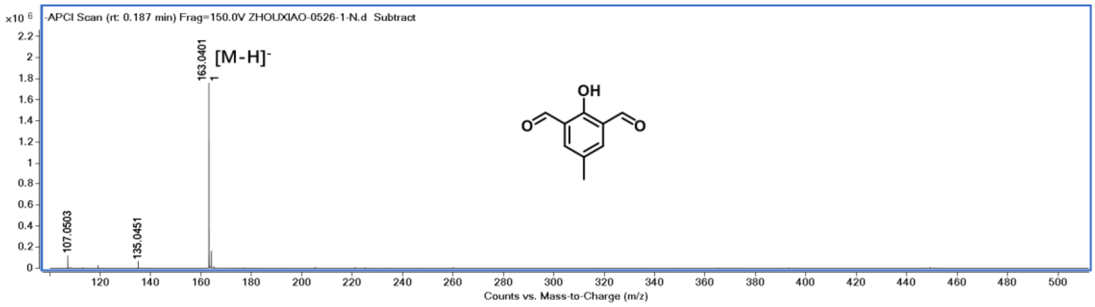

Figure S24. ESI-MS spectrum of 2a.

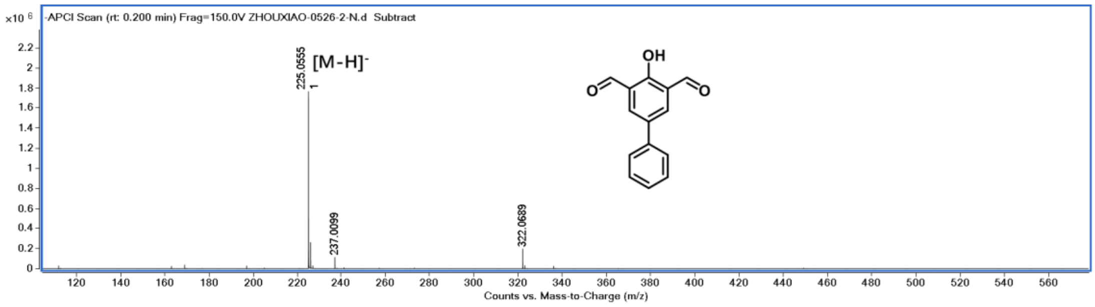

Figure S25. ESI-MS spectrum of 2b.

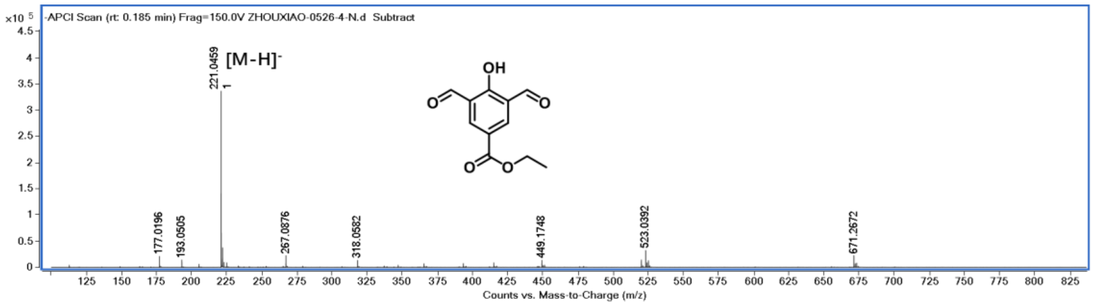

Figure S26. ESI-MS spectrum of 2c.

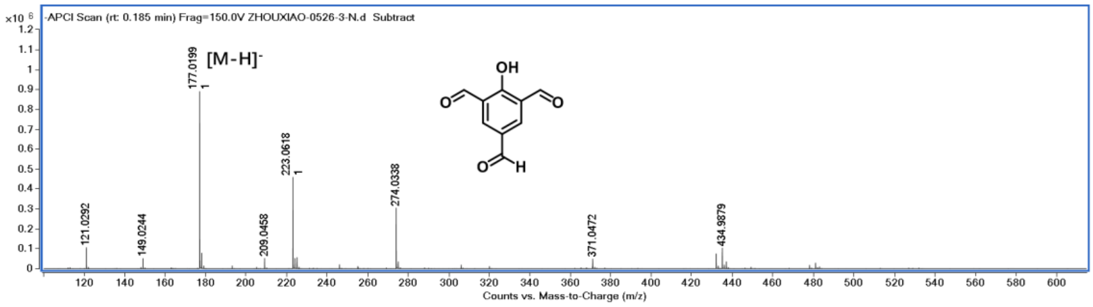

Figure S27. ESI-MS spectrum of 2d.

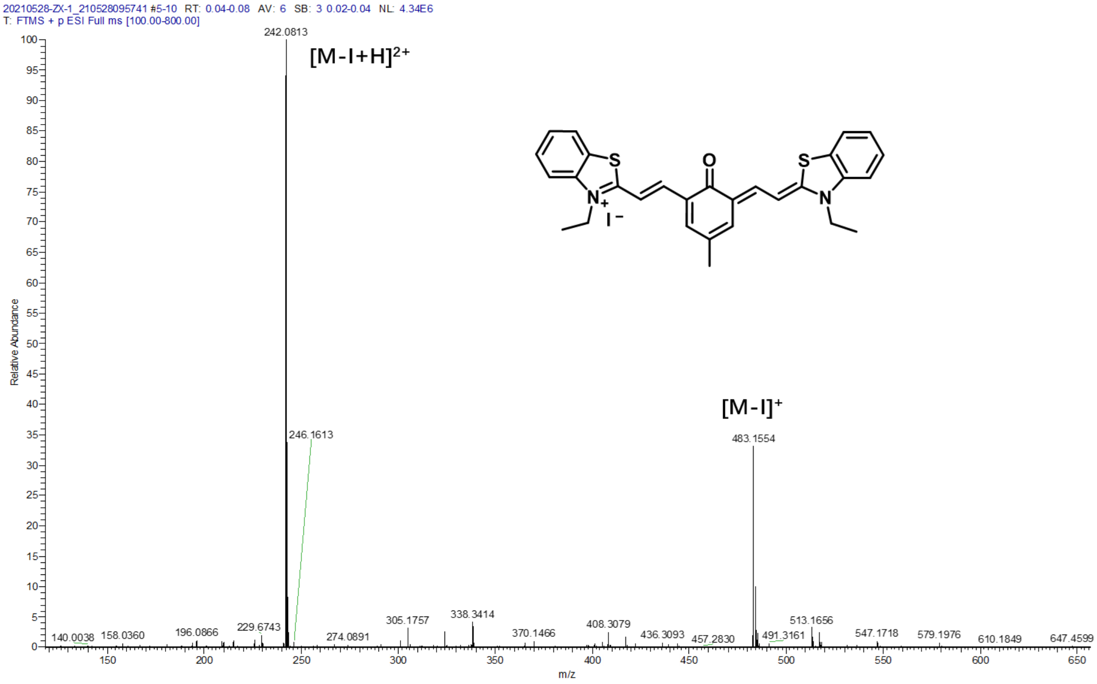

Figure S28. ESI-MS spectrum of QTCy7-Me.

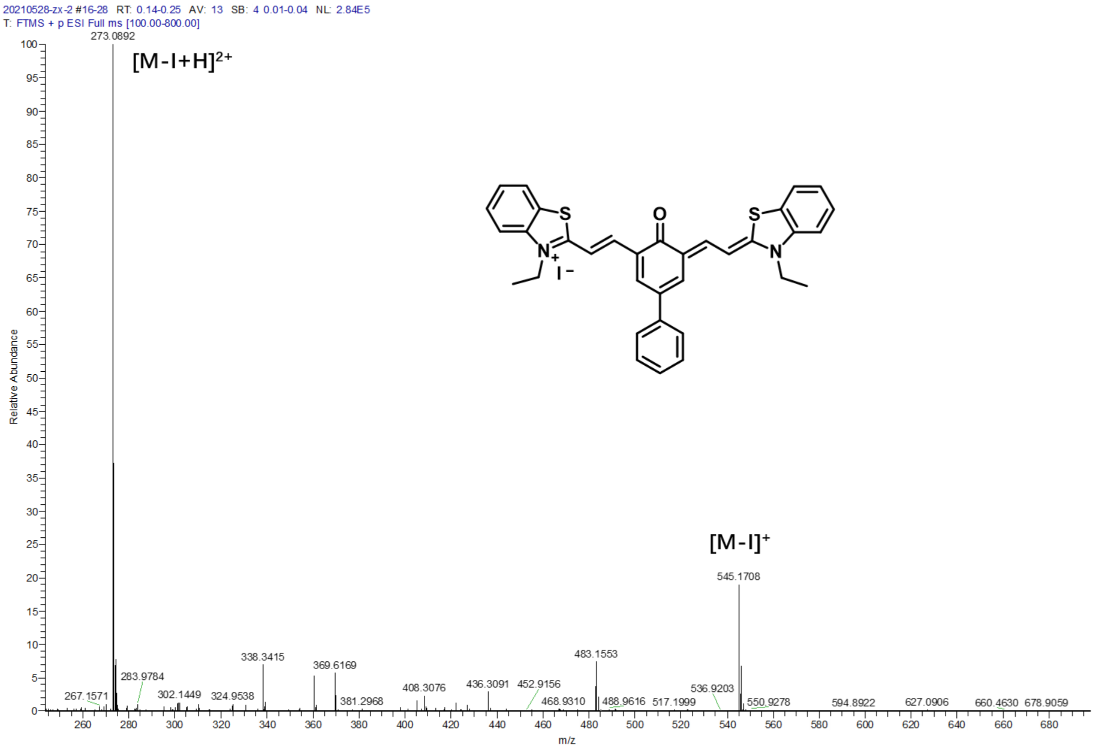

Figure S29. ESI-MS spectrum of QTCy7-Ph.

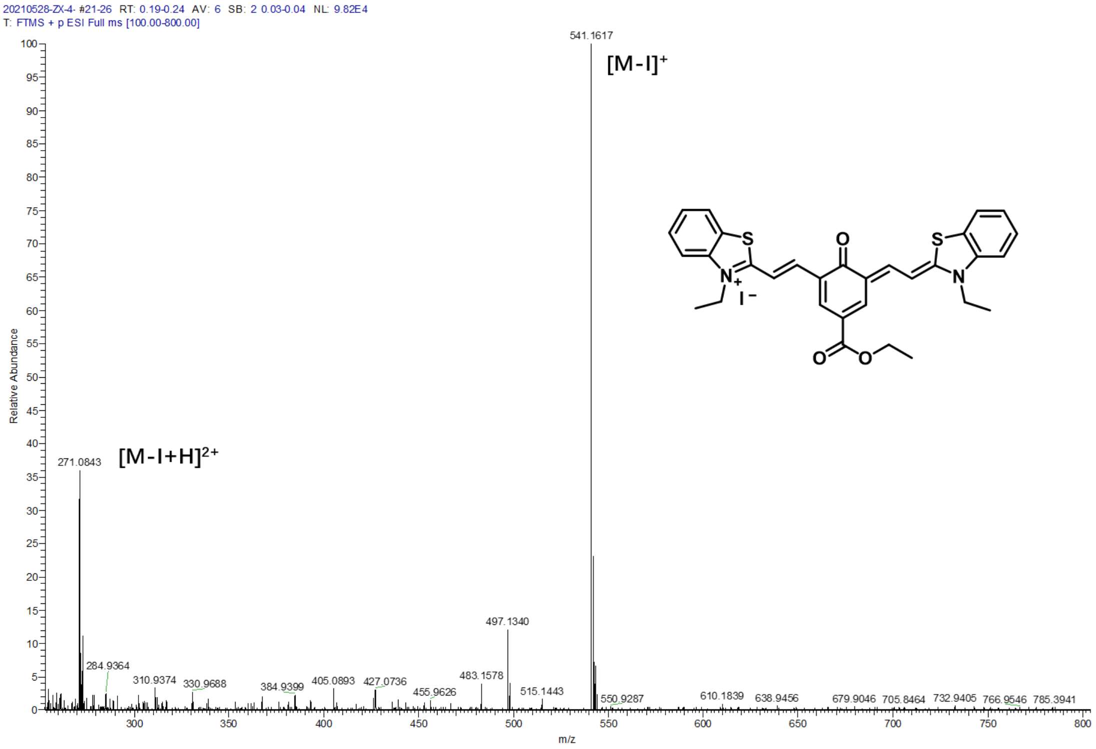

Figure S30. ESI-MS spectrum of QTCy7-Ac.

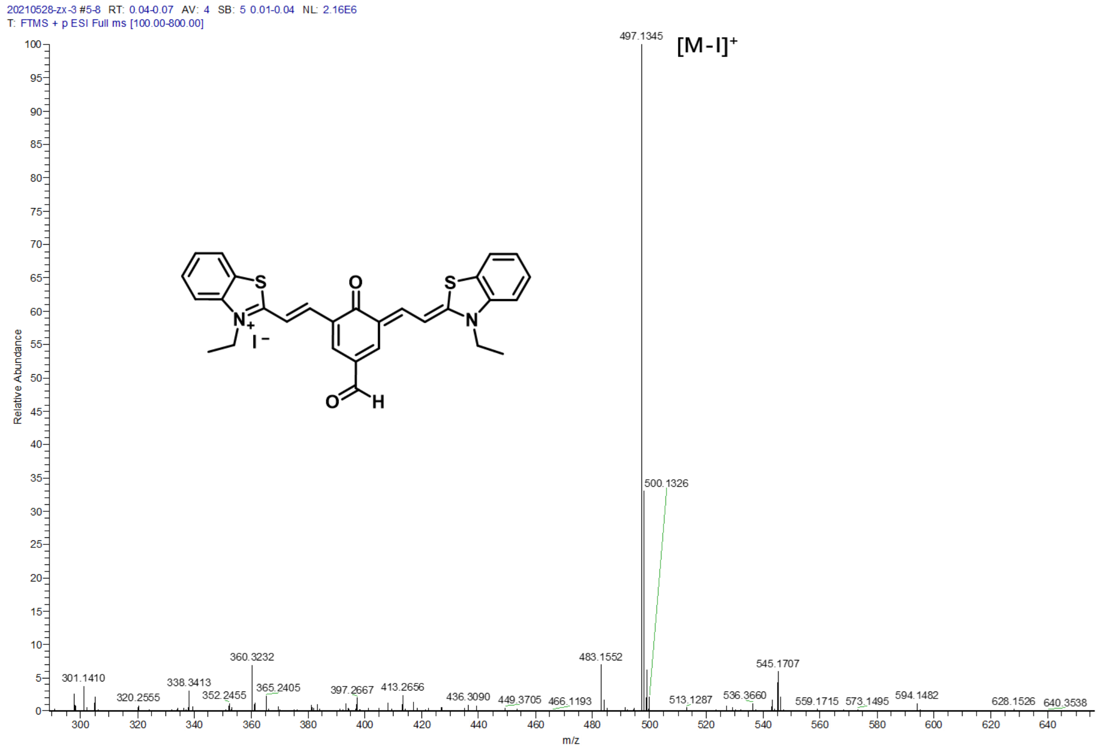

Figure S31. ESI-MS spectrum of QTCy7-CHO.

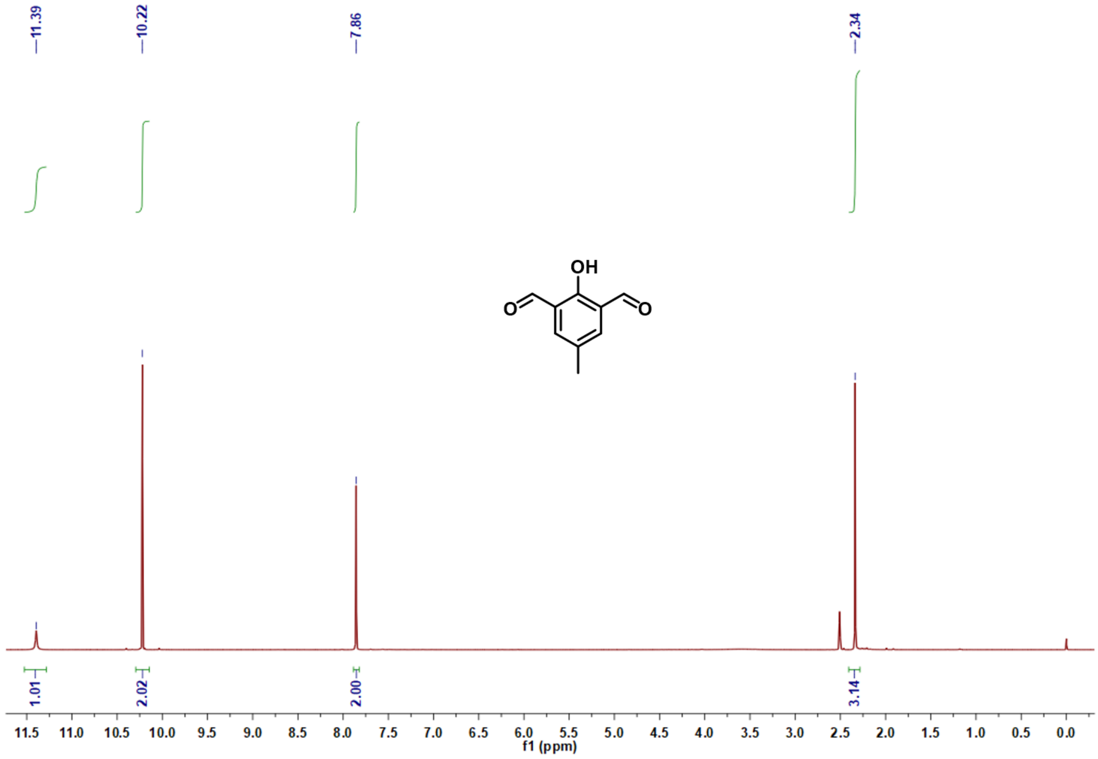

Figure S32. ^{1}H NMR spectrum of 2a.

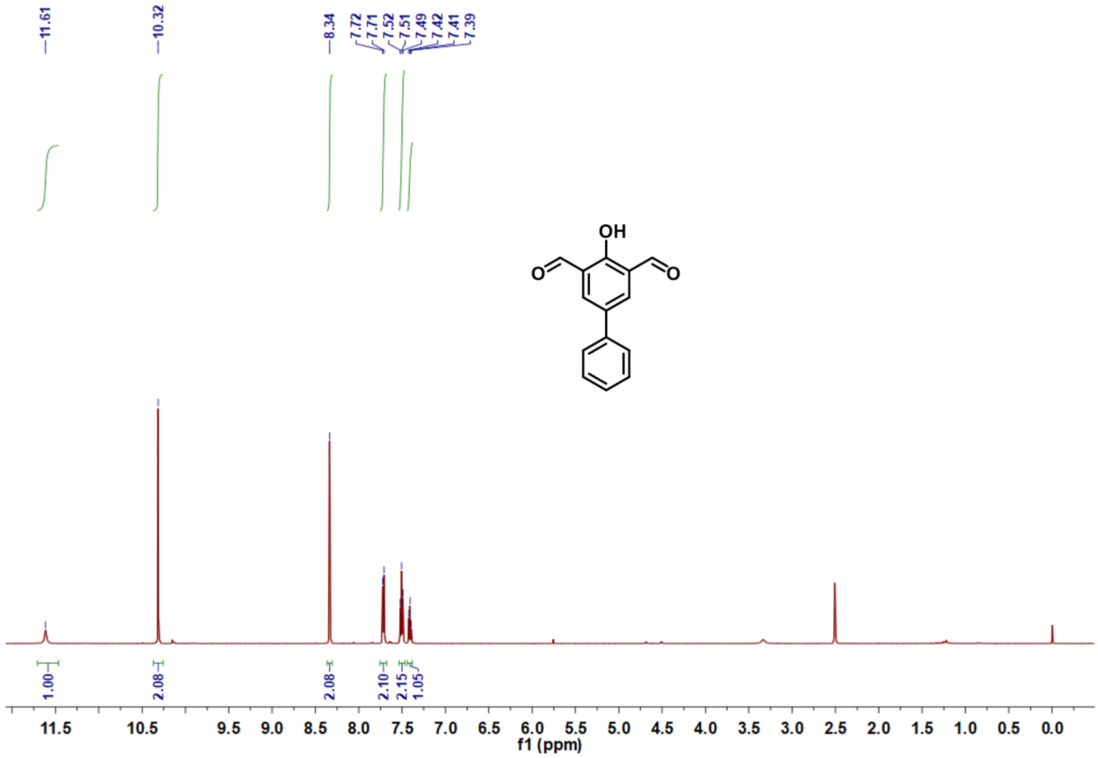

Figure S33. ^{1}H NMR spectrum of 2b.

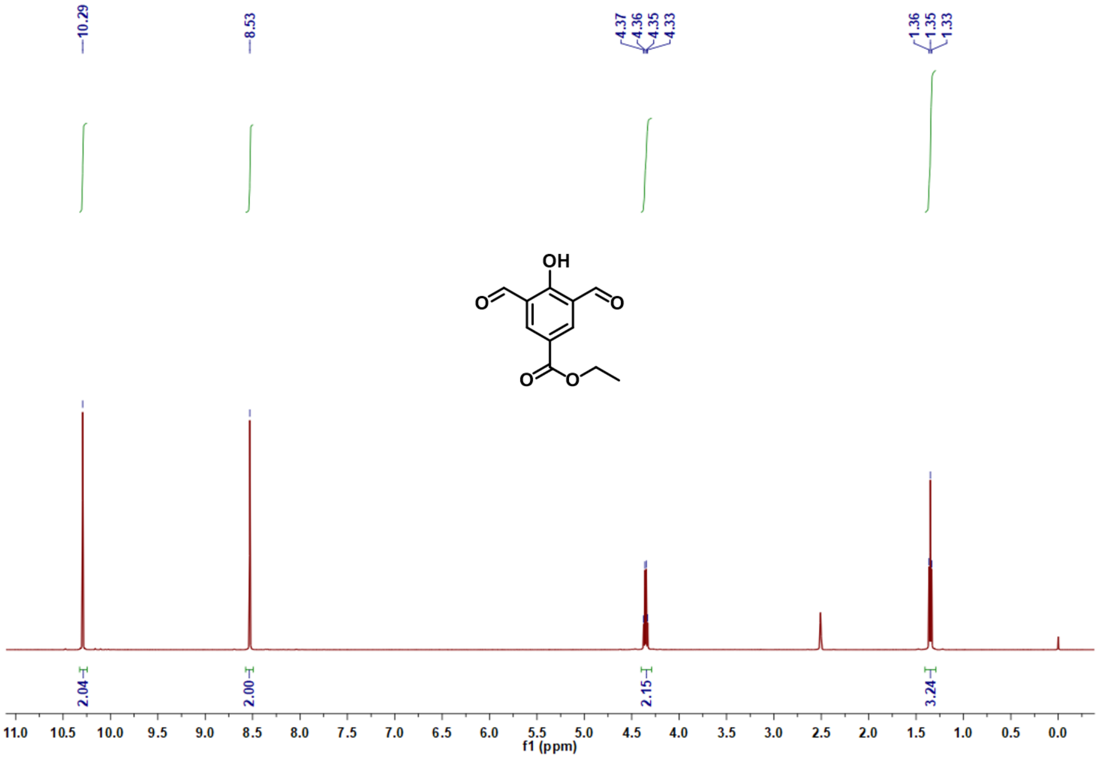

Figure S34. ^{1}H NMR spectrum of 2c.

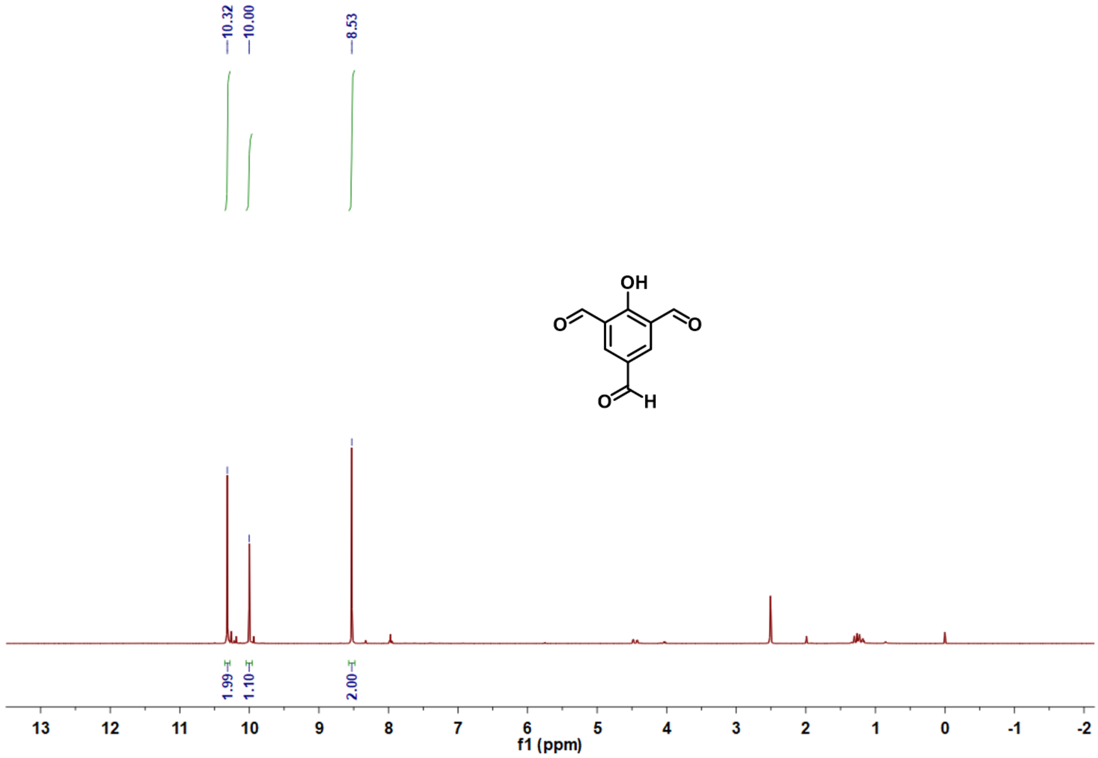

Figure S35. ^{1}H NMR spectrum of 2d.

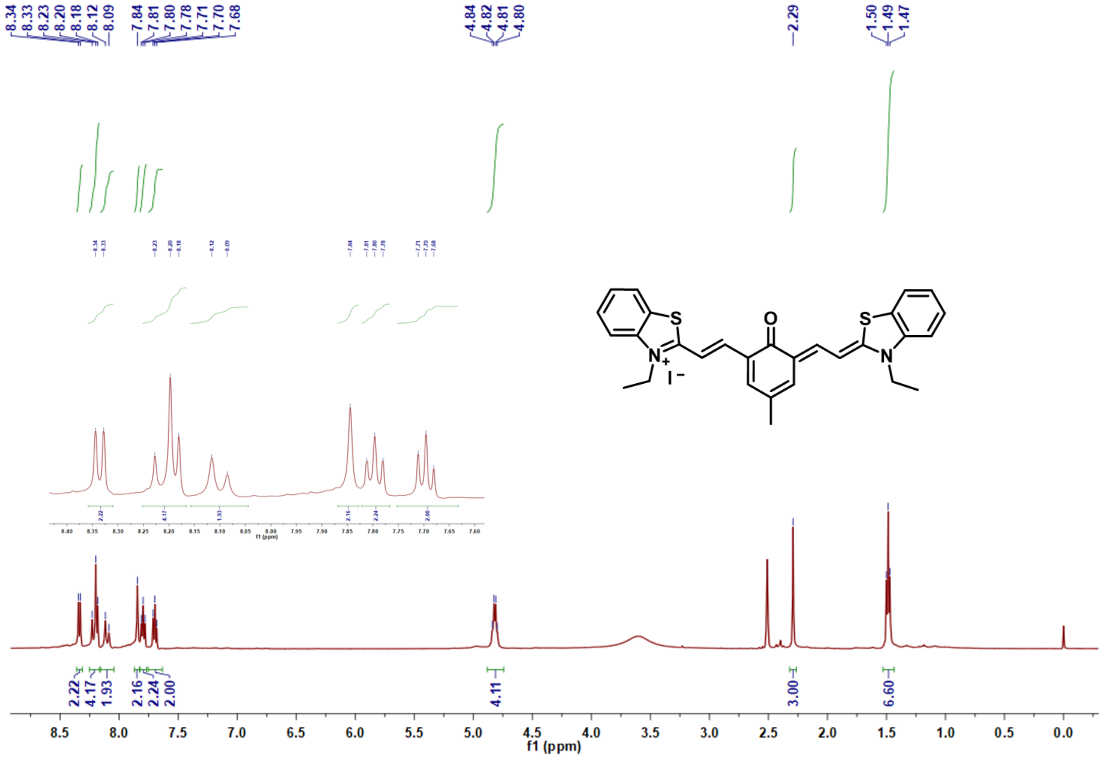

Figure S36. ^{1}H NMR spectrum of QTCy7-Me.

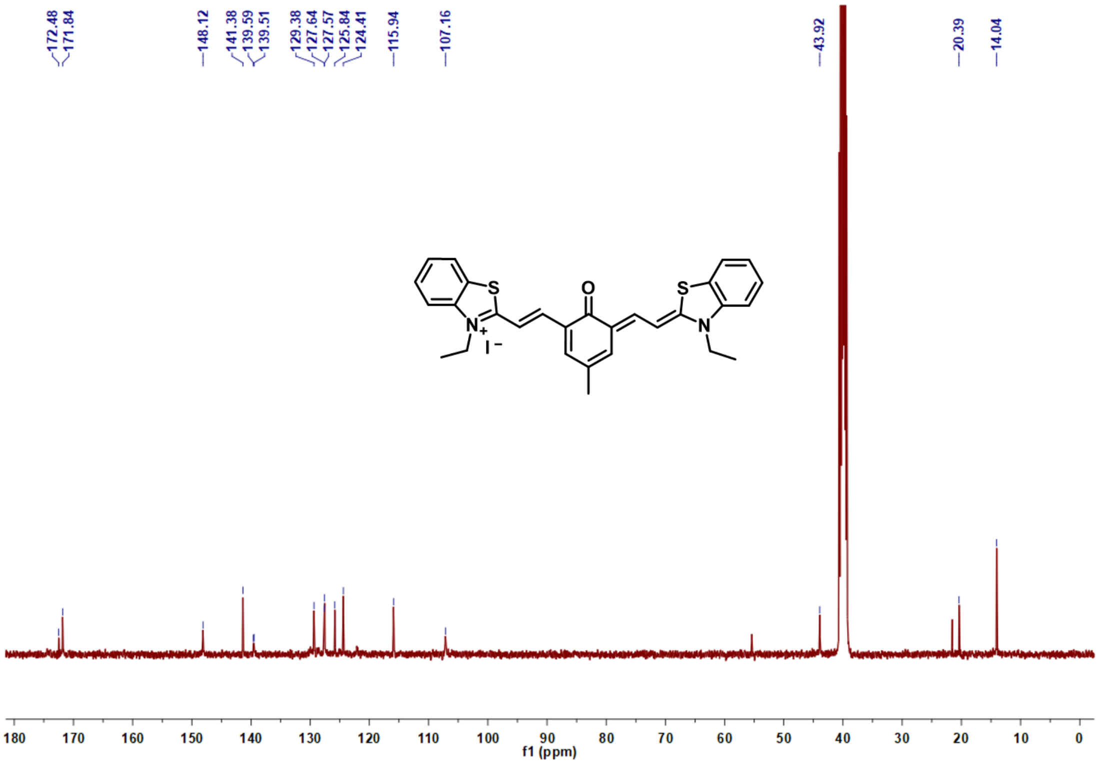

Figure S37. ^{13}C NMR spectrum of QTCy7-Me.

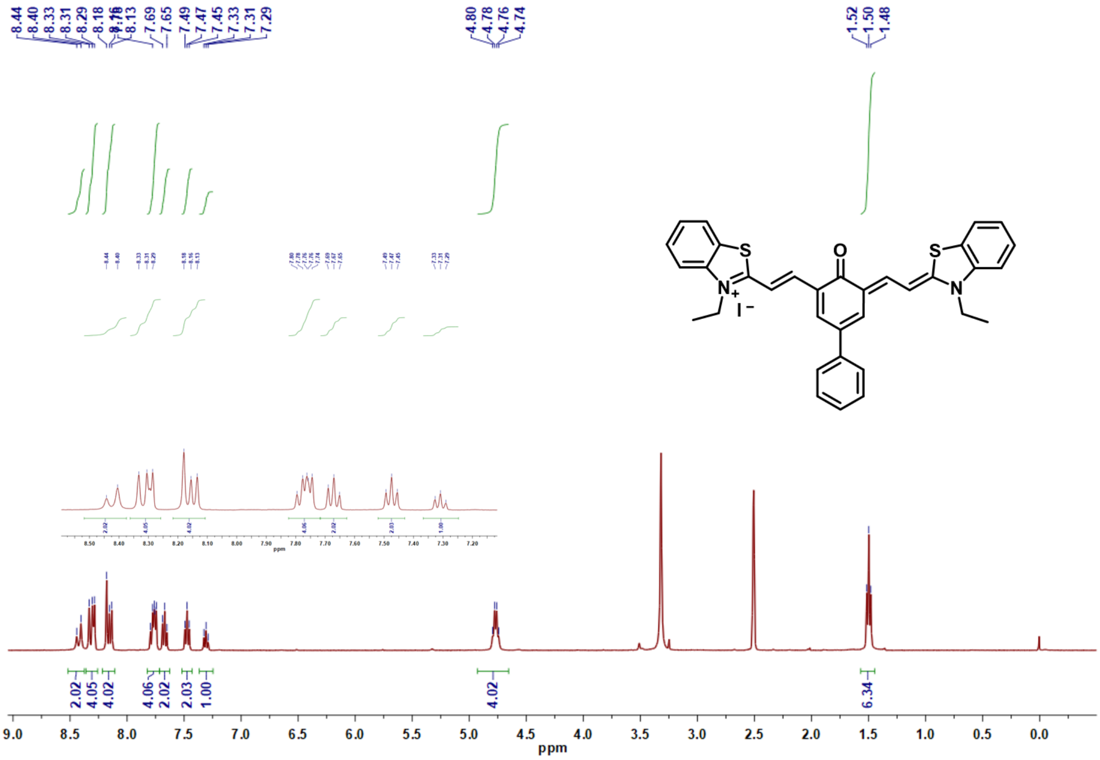

Figure S38. ^{1}H NMR spectrum of QTCy7-Ph.

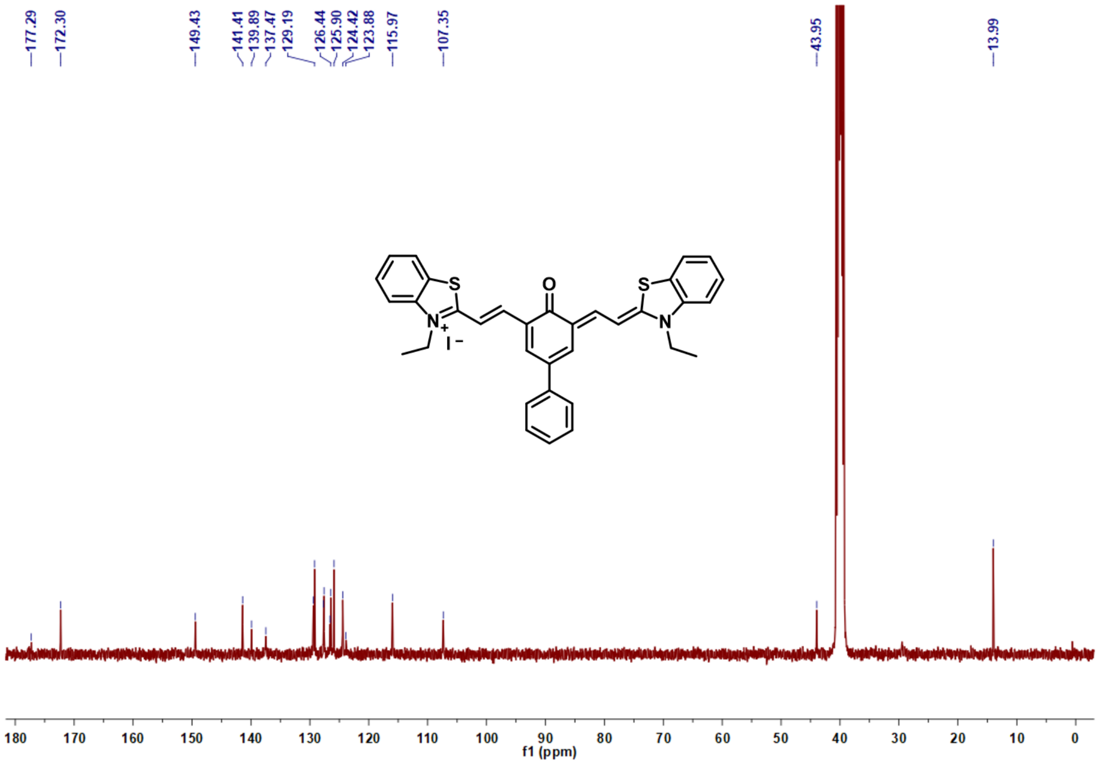

Figure S39. ^{13}C NMR spectrum of QTCy7-Ph.

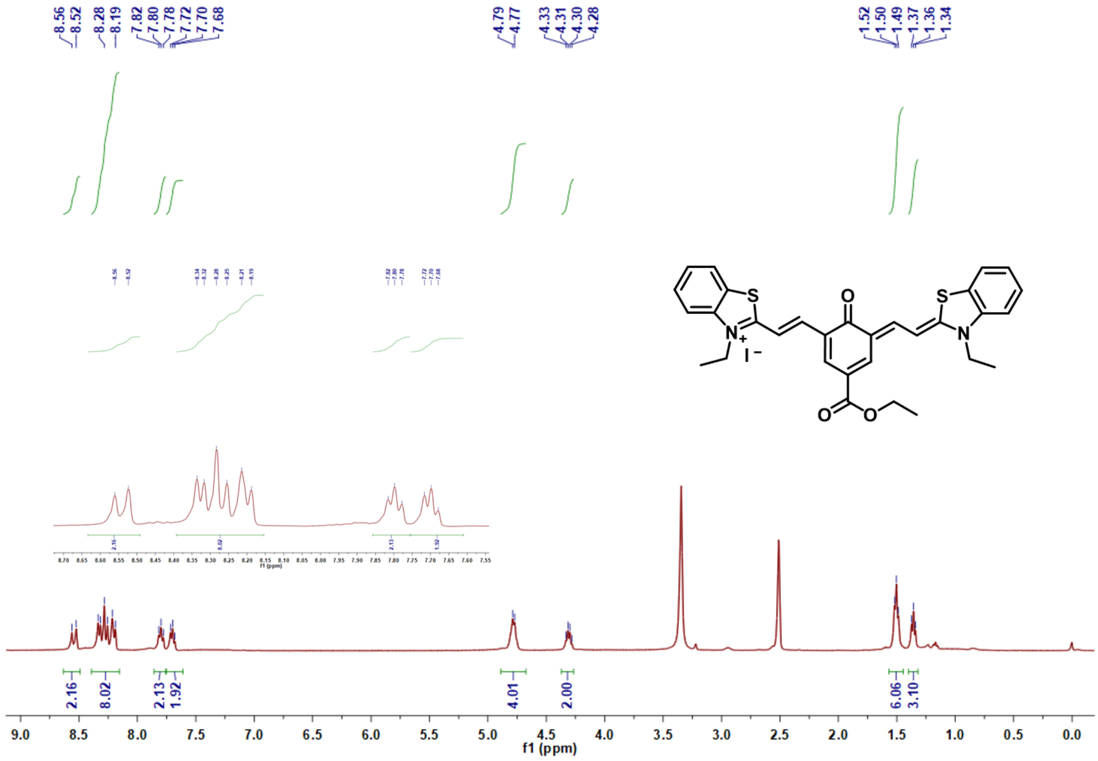

Figure S40. ^{1}H NMR spectrum of QTCy7-Ac.

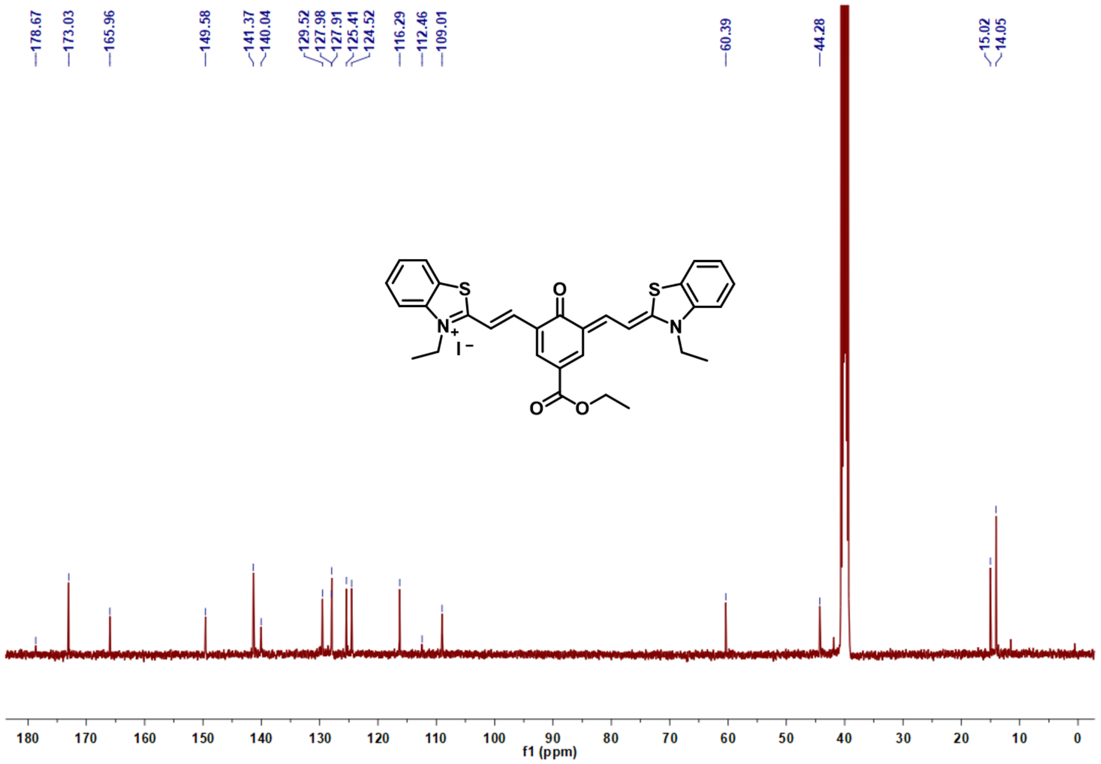

Figure S41. ^{13}C NMR spectrum of QTCy7-Ac.

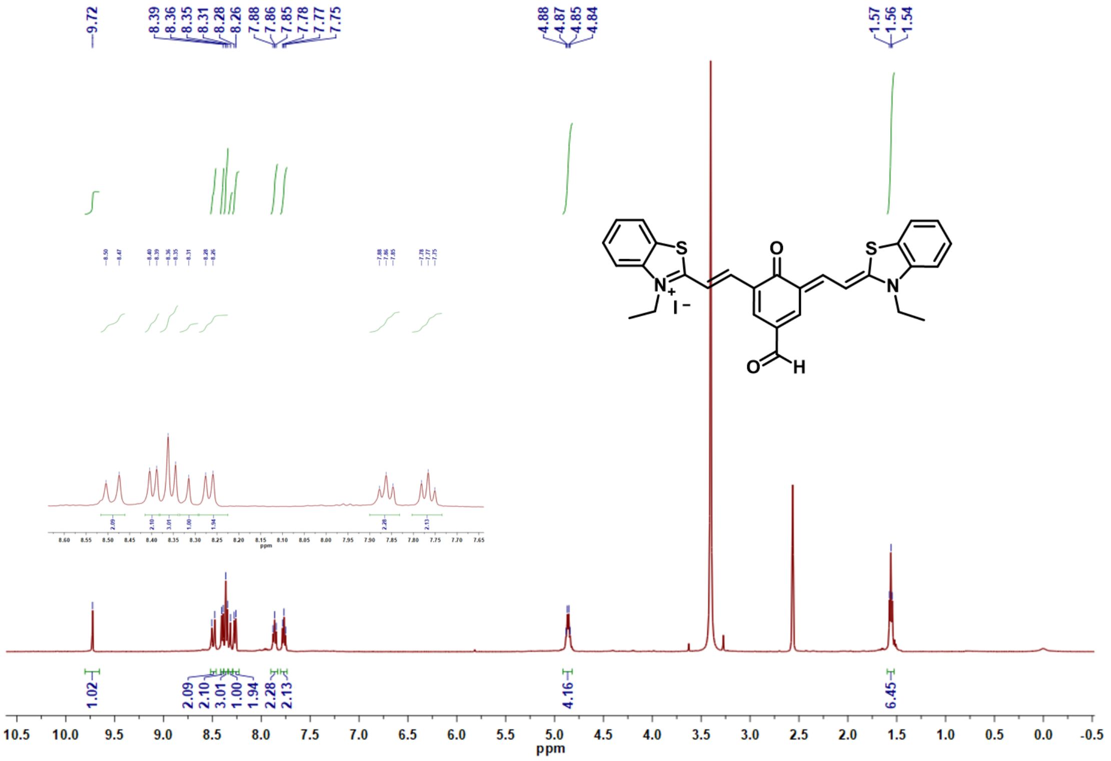

Figure S42. ^{1}H NMR spectrum of QTCy7-CHO.

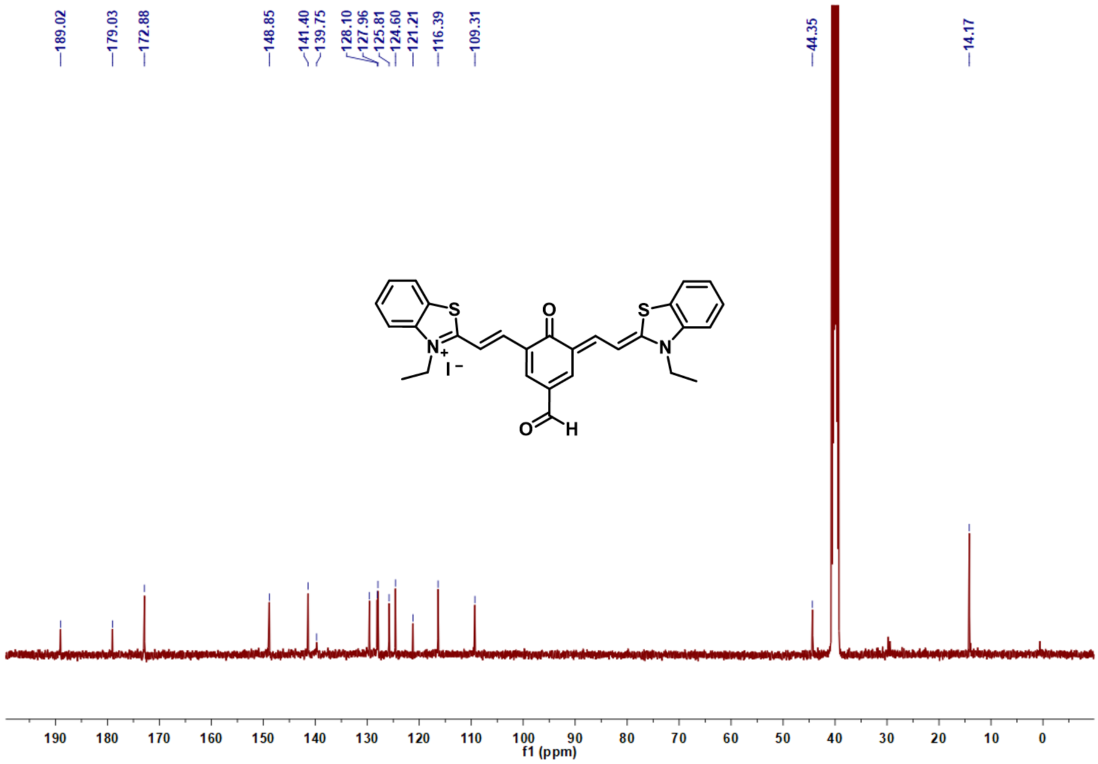

Figure S43. ^{13}C NMR spectrum of QTCy7-CHO.

References

(1) Ma, H.; Lu, Y.; Huang, Z.; Long, S.; Cao, J.; Zhang, Z.; Zhou, X.; Shi, C.; Sun, W.; Du, J.et al. ER-Targeting Cyanine Dye as an NIR Photoinducer to Efficiently Trigger Photoimmunogenic Cancer Cell Death. *J. Am. Chem. Soc.* **2022**, *144*, 3477-3486.

(2) Zhao, X.; Long, S.; Li, M.; Cao, J.; Li, Y.; Guo, L.; Sun, W.; Du, J.; Fan, J.; Peng, X. Oxygen-Dependent Regulation of Excited-State Deactivation Process of Rational Photosensitizer for Smart Phototherapy. *J. Am. Chem. Soc.* **2020**, *142*, 1510-1517.

(3) Neese, F. Software update: the ORCA program system, version 4.0. *WIREs Computational Molecular Science* **2018**, *8*, e1327.

(4) Lu, T.; Chen, F. Multiwfn: A multifunctional wavefunction analyzer. *J. Comput. Chem.* **2012**, *33*, 580-592.

(5) Štacková, L.; Muchová, E.; Russo, M.; Slavíček, P.; Štacko, P.; Klán, P. Deciphering the Structure–Property Relations in Substituted Heptamethine Cyanines. *J. Org. Chem* **2020**, *85*, 9776-9790.
